# Evidence of organized but not disorganized attachment in wild Western chimpanzee offspring (*Pan troglodytes verus*)

**DOI:** 10.1038/s41562-025-02176-8

**Published:** 2025-05-12

**Authors:** Eléonore Rolland, Oscar Nodé-Langlois, Patrick J. Tkaczynski, Cédric Girard-Buttoz, Holly Rayson, Catherine Crockford, Roman M. Wittig

**Affiliations:** 1 Ape Social Mind Lab, Institut des Sciences Cognitives Marc Jeannerod, University of Lyon (CNRS UMR 5229), Bron, France; 2 Department of Human Behavior, Ecology and Culture, Max Planck Institute for Evolutionary Anthropology, Leipzig, Germany; 3 Tai Chimpanzee Project, Centre Suisse de Recherches Scientifiques en Côte d’Ivoire, Cote d’Ivoire, Abidjan, Côte d’Ivoire; 4 Université Claude Bernard Lyon 1, Villeurbanne, France; 5 Research Centre for Evolutionary Anthropology and Paleoecology, School of Biological and Environmental Sciences, Liverpool John Moores University, Liverpool, UK; 6 ENES Bioacoustics Research Laboratory, Centre de Recherche en Neurosciences de Lyon, University of Saint-Etienne (CNRS, Inserm), Saint-Etienne, France; 7 Institut des Sciences Cognitives Marc Jeannerod, University of Lyon 1 (CNRS UMR 5229), Bron, France

**Keywords:** Animal behaviour, Human behaviour, Social anthropology

## Abstract

Human attachment theory outlines three organized types: secure, insecure avoidant and insecure resistant, all considered adaptive responses to maternal care for offspring survival. In contrast, disorganized attachment is hypothesized to be maladaptive and therefore uncommon in wild mammals, though this remains untested. We assessed attachment types in 50 wild chimpanzees (ages 0–10 years) in Taï National Park, Côte d’Ivoire. Using 3,795 h of mother and offspring focal observations, we found no behaviours indicative of disorganized attachment. To explore organized attachment, we analysed a subset of 18 immature chimpanzees and their behavioural responses to 309 natural threatening events. Their responses showed organized attachment patterns: some sought maternal closeness (secure-like), while others displayed independence (insecure avoidant-like). Our study supports the hypothesis that organized attachment types are adaptive and have a long evolutionary history.

## Abstract (web summary)

This study of 50 wild Western chimpanzee mother–offspring dyads revealed no evidence of disorganized attachment. Instead, offspring exhibited secure-like and insecure avoidant-like behaviours during threats, consistent with the theory that attachment is an adaptive trait.

## Main

Attachment is defined as an affectionate bond between an infant and primary caregiver, characterized by physical proximity and reactions to separation^1,2^. In humans, attachment is crucial for both the physical and psychological well-being of the offspring^3^. Attachment theory suggests that the caregiver, usually the mother, serves as a secure base, enabling exploration and providing a safe haven for the infant during distress^4^.

**Fig. 1:**
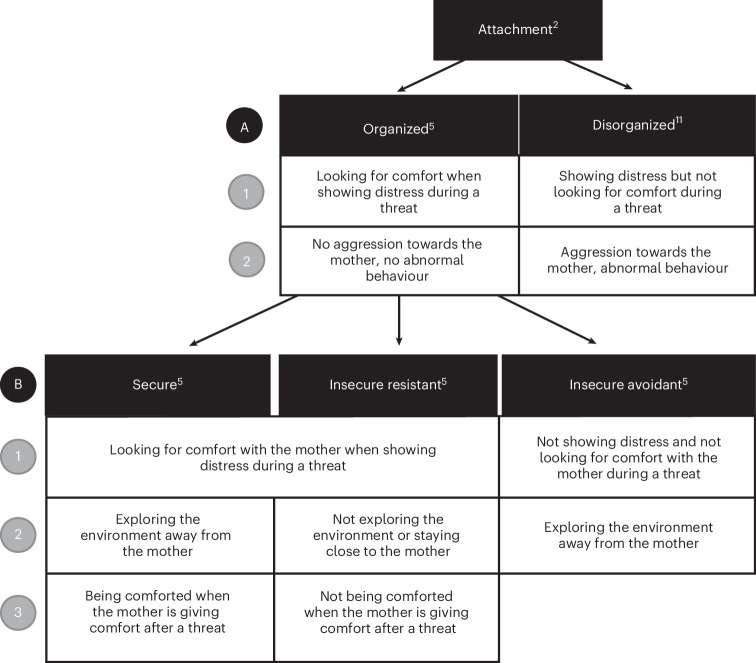
Representation of the different types of attachment as defined in humans and our predictions to determine whether these exist in natural chimpanzee societies. The black boxes show human attachment types. The white boxes depict the respective predicted behaviour of offspring towards mothers, given the applied assessments. The letters on the left refer to the different types of attachment. The numbers on the left refer to the analyses to test the predictions related to the different types of attachment in the Methods and the Results sections. The numbers indicated in superscript correspond to the references in the literature: Bowlby^2^, Ainsworth et al.^5^ and Main and Solomon^11^.

In humans, offspring attachment to a primary caregiver is commonly examined using an experimental paradigm known as the Strange Situation Procedure (SSP)^5,6^. According to the SSP, different types of attachment can be classified based on the assessment of the offspring’s perception of safety in the caregiver–offspring relationship. This is defined as confidence in the caregiver’s availability and responsiveness, and is revealed by exposing the child to a temporary separation from their caregiver. Ainsworth and colleagues^5^ described three types of ‘organized’ attachment, referring to attachment behaviours that are structured and consistent: (1) secure, (2) insecure resistant/ambivalent and (3) insecure avoidant (Fig. 1). Organized attachment is hypothesized to represent strategies adapted to the caregiver’s responsiveness, ensuring offspring survival and sustained development in diverse early social settings. Such strategies are thought to be formed to optimize social capacity in the relevant environment^2,7^. Secure attachment arises from confidence in the caregiver’s availability, nurtured by their high responsiveness. On the other hand, insecure-avoidant offspring adjust their behaviour due to their caregiver’s lack of responsiveness, leading to avoidance of contact during distress and developing a level of independence earlier than is typical for their age. Insecure-resistant offspring exhibit clingy behaviour, a response to the inconsistent responsiveness of their caregiver. Their inability to venture away from their mothers serves as an indicator of their attachment security^8^. Research shows that attachment types remain stable across development and influence social growth. Waters^9^ found consistent infant behaviour during the SSP between 12 and 18 months. Kerns, Tomich and Kim^10^ used questionnaires to reveal that children aged 7–13 years maintained stable perceptions of attachment figure availability (with no change in the secure score for parent–child attachment), though they relied less on them as they aged, seeking less comfort or play from caregivers when distressed or sick.

Main and Solomon^11^ introduced a fourth attachment type, insecure-disorganized/disoriented attachment (Fig. 1), characterized by random or conflicting behaviours, stereotypies and fear of the caregiver typically, with offspring expressing distress without seeking comfort from their caregiver^12,13^. Disorganized attachment, linked to problematic parenting and higher rates of parental psychopathology, poses challenges for social integration^14^. The classification of disorganized attachment as adaptive^2,15^ or maladaptive^16^ remains debated. Indeed, Gazzillo and colleagues^15^ consider disorganized attachment as a child’s adaptation to a traumatic, inconsistent environment, leading to contradictory behaviours such as avoiding approach and showing fear. Other researchers, in contrast, argue that disorganized attachment is maladaptive, lacking a coherent strategy and posing long-term developmental risks^16,17^. Understanding the evolutionary history of these behaviours could offer insights into child development, aiding therapeutic interventions and prevention strategies for emotional and psychological issues.

Unlike all other apes, reproduction in humans is relatively cooperative and includes a range of support structures that theoretically could limit the impact of poor parent–offspring attachment^18^. As such, disorganized attachment could emerge as a maladaptive response to disorganized parenting, which could arise from a lack of clear selection pressures on parenting. In this case, disorganized attachment is unlikely to be observed in environments where natural selection should favour more adaptive attachment patterns that increase the chances of offspring survival. Therefore, as a first step towards evaluating the evolution of certain attachment types, a comparative approach is needed.

Attachment theory is proposed to apply broadly to mammals to aid offspring survival^19^, but so far, it has predominantly been applied to human relationships. However, researchers have explored the mother–offspring relationship in captive nonhuman primates. Such studies demonstrate strong attachment bonds between biological mothers and their infants^20^. Furthermore, studies with monkeys, including Japanese macaques (*Macaca fuscata*)^21^, bonnet macaques (*Macaca radiata*)^22^, brown capuchin monkeys (*Cebus apella*)^23^ and rhesus macaques (*Macaca mulatta*)^24^, have identified individual differences in mother–offspring attachment security. Additionally, Yano-Nashimoto and colleagues^25^ found that captive infant marmosets (*Callithrix jacchus*), which are raised in a multi-caregiver system, selectively avoided and emitted negative calls when handled by rejecting caregivers. This suggests attachment figures are chosen depending on the quality of care provided in this species.

Studies of mother–offspring attachment types in wild primates, however, have not yet been conducted. Chimpanzees, one of our closest living relatives^26^, serve as an excellent model for studying mother–offspring attachment. Their exceptional social and cognitive abilities, coupled with an important dependence on mothers for at least the first decade of life^27–30^, make them extremely valuable for comparative research. This social dependency crucially contributes to the offspring’s overall fitness^31,32^. Van Ijzendoorn et al.^33^ and Clay^34^ used the SSP with captive human-reared infant chimpanzees, revealing distinct attachment types with their favourite caregiver similar to those in human infants with their mothers. Responsive care (as opposed to standard care) correlated with less disorganized attachment and more advanced cognitive development. One-year-old captive chimpanzees with disorganized attachment had an increased likelihood of illness, heightened abnormal behaviours and reduced social interaction success over 20 years (ref. ^34^).

While insightful, findings from such studies potentially differ from those representing mother–offspring attachment in natural settings. Mothers not only provide nourishment, but also closeness, comfort and social learning opportunities^35^. While captive settings provide a more controlled environment, it is essential to acknowledge their limitations in representing attachment processes in a natural system where behaviours are largely adapted to socio-ecological conditions. Owing to predation, social competition and variable food availability, mother–offspring attachment under natural conditions should be shaped by selection processes.

In our study of wild chimpanzees, we first hypothesized that disorganized attachment would be rare in these natural settings due to its potentially maladaptive nature in humans, with some evidence supporting this in captive apes^34^. Given that disorganized attachment may result, at least in part, from the maternal style the offspring is exposed to, low survival rates would limit the proliferation of such a behavioural phenotype into future generations. We predicted that immature chimpanzees would show few behaviours typically observed in individuals with disorganized attachment. Specifically, we predicted immature chimpanzees would show few aggressive behaviours towards their mother, rare or no abnormal or stereotypic behaviour and would look for comfort when expressing vocal distress. Second, we hypothesized that organized attachment types would be prevalent in wild chimpanzees. In humans, caregiver responsiveness is thought to directly shape the attachment type^5,36,37^, but this has not been tested in nonhuman animals. Nevertheless, general social characteristics such as gregariousness, grooming rates and number of bond partners vary among chimpanzee mothers^38^ and depend on the sex of the offspring^39^, which might impact maternal care. Therefore, we expected some variation in attachment type in chimpanzee offspring. We compared attachment patterns with existing and well-defined attachment types observed in humans. We expected that if attachment types overlap with those of humans, wild immature chimpanzees would display different safety-seeking and comfort behaviours from mothers during threatening situations, reflecting secure, insecure-avoidant and insecure-resistant attachment types. Additionally, to assess the functional similarity of any attributed attachment types with those of humans, we determined how attachment security impacted mother–offspring proximity during social exploration^8^. We predicted that individuals who did not rely on their mother during exposure to a threat (insecure avoidant-like), would explore further away from her during social contexts, compared with those seeking comfort during threatening events (secure and insecure resistant-like). We also expected that individuals exhibiting overreaction during threatening situations (insecure resistant-like) would limit their exploration in social contexts, staying close to their mother compared to other individuals. The analyses performed to test each prediction are summarized in Fig. 1.

Therefore, the current research endeavoured to investigate the dynamics of mother–offspring attachment in wild western chimpanzees (*Pan troglodytes verus*), drawing parallels with attachment theory in humans. The behavioural data (ethogram in Supplementary Table 1) collected over 34 months (from 2016 to 2023) on 50 mother–offspring dyads (offspring’s age between 0 and 10 years old) (Supplementary Table 2) provides a unique opportunity to explore the relevance of attachment theory in chimpanzees within their natural habitat. This approach also offers a fresh perspective by studying attachment types in wild primates.

## Results

To investigate mother–offspring aggression (test A2) and test for disorganized attachment, we used the entire dataset (Supplementary Fig. 1). For analyses related to organized attachment (test B) and part of the disorganized attachment assessment (test A1), we focused on data from 2021 to 2023, which included additional ethogram elements specifically designed to capture responses to directed and undirected threatening events (UTEs) (see ‘Threatening events’ section) (Supplementary Fig. 1).

We collected 2,882 h of behavioural data on 50 immature chimpanzees (22 females and 28 males, aged 0 to 10years, mean observation time (*M*) = 58.06 h, range 28–107 ± 19.96 h (s.d.) per individual), and 913 h on 21 mothers to assess maternal aggressiveness (*M* = 36.63 h, range 12–58 ± 10.67 h (s.d.) per individual), totalling 3,795 h of observation.

### Testing the existence of disorganized attachment

#### Comfort seeking during distress

To investigate the existence of disorganized attachment in 30 immature chimpanzees (ages 0 to 10 years), we tested whether vocal distress from the offspring predicted a mother–offspring approach during naturally occurring events (Model 1), a sign of organized rather than disorganized attachment. We categorized these events as undirectly or directly threatening for the offspring UTEs and directed threatening events (DTEs)). Whimpering or screaming predicted the likelihood of the mother–offspring approach (Table 1). Offspring showing vocal distress (whimpering or screaming) approached their mother (23% of 168 occasions) or were approached by her (42% of 168 occasions). We examined the credible intervals (CIs) for each individual to identify individuals not following the predictions of the model (Supplementary Fig. 2). We found that vocal distress did not predict an approach for three individuals out of 30 (10%). By examining the behavioural reactions during other threatening events, we found that these three individuals approached their mother on several occurrences. The conditional *R*^2^ of this model was 0.284.

**Table 1:** Vocal distress predicting mother–offspring approaches during threatening events (Model 1)

Predictors	Estimate	Estimate error	95% CI	89% CI
Intercept	−0.73	0.64	(−2.01 to 0.48)	(−1.77 to 0.26)
Whimper or scream	0.84	0.34	**(0.19 to 1.52)**	**(0.32 to 1.39)**
Offspring age	−0.77	0.28	**(−1.31 to −0.22)**	**(−1.21 to −0.33)**
Party size	−0.14	0.13	(−0.39 to 0.11)	(−0.34 to 0.06)
Sex	0.45	0.48	(−0.49 to 1.39)	(−0.31 to 1.21)
Group East	−0.35	0.60	(−1.53 to 0.83)	(−1.32 to 0.61)
Group South	−0.52	0.57	(−1.62 to 0.61)	(−1.42 to 0.40)

The 95% CI and 89% CI are the CIs at their respective confidence levels. The reference levels for each predictor are no whimper nor scream for ‘Whimper or scream’, female for ‘Sex’ and North for ‘Group’. The numbers in bold represent CIs excluding 0. The sample size is *n* = 30 individuals across 567 independent events.

#### Aggression between mother and offspring

We assessed the occurrence of aggressive behaviours between immature chimpanzees and their mother and other characteristic behaviours of disorganized attachment to investigate the existence of this attachment type. In over 3,795 h of focal data focusing on mother and offspring, the 50 studied offspring between 0 to 10 years old never showed aggression towards the mother. In contrast, we observed a total of 31 mild non-contact aggressions of 12 mothers towards their offspring during the observation period (27 arm waves, 2 hunches, 1 charge and 1 chase) and 15 contact aggressions of 11 mothers towards their offspring (6 push away, 6 hits and 3 pulls). Mother–offspring aggression rates are shown in Supplementary Fig. 3.

We did not observe any abnormal or stereotypic behaviours in any chimpanzee (for example, rocking, hair pulling, face grimacing or incomplete behaviour), nor attempts by the offspring to escape from their mother, except in instances where the mother displayed aggression towards the offspring over the 50 studied offspring.

### Existence of different organized attachment types

#### Reactions of offspring during threatening events

**Fig. 2:**
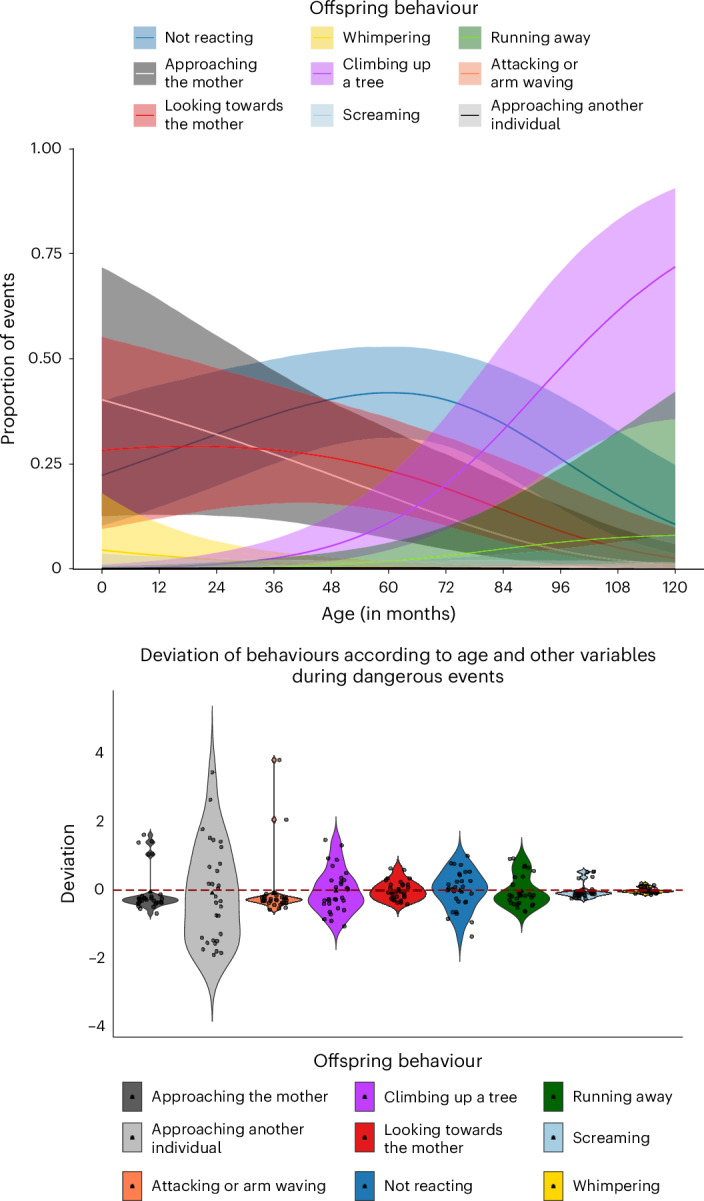
Offspring behavioural reactions to UTE. Top: the likelihood of a particular behavioural reaction by the offspring when experiencing a UTE, in relation to the offspring’s age.The solid lines depict the proportion of events in which each behaviour was observed and predicted by the model, and the coloured areas are the 95% CI. Bottom: individual variation in offspring behavioural reactions to UTEs. The violin plots show deviation from the expected value (0) for a given age while accounting for sex, party size and the presence of older siblings. Each dot depicts an individual chimpanzee. Positive or negative deviation from zero indicates that the individual shows a behaviour more or less than predicted, respectively. The sample size is *n* = 30 individuals across 550 independent events.

We investigated the behavioural reactions of 18 immature chimpanzees during UTEs and found differences across ages. The youngest chimpanzees (less than 2 years) predominantly whimpered, looked towards their mother and/or approached their mother during a UTE (model 2; Fig. 2a). However, with increasing age, this tendency decreased. The tendency to show no reaction increased with age and peaked at 50% of events around the weaning period (4–6 years old) before the likelihood of no reaction decreased again. After weaning (>6 years), however, the likelihood of climbing a tree or running away from the UTE increased.

Comparing the deviation estimates of each offspring’s behaviour when reacting to the UTE with the expected behaviour for its age and other variables (for example, sex or presence of older siblings), we found that some behavioural reactions had higher individual variability than others (Fig. 2b). Offspring showed different interindividual variability across the behaviours, with the highest variability for the behaviour ‘approaching the mother’ (deviation of 4.78), all the other behaviours had a deviation under 2.3 indicating lower variability. This suggests that interindividual variability in behavioural reactions during UTEs was primarily influenced by differences in the approach behaviour towards the mother.

**Fig. 3:**
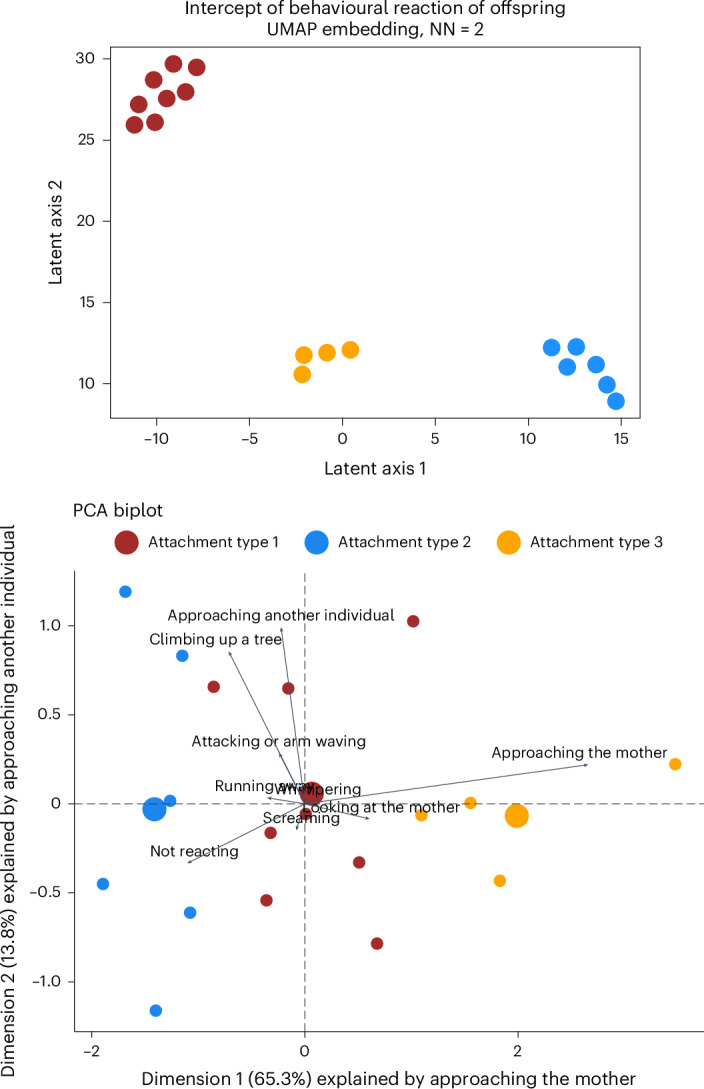
Interindividual variation of offspring reactions during UTE (results of the UMAP, cluster analysis and PCA). Each dot corresponds to an individual offspring. Top: dimension reduction (UMAP) and cluster analysis of the behavioural reactions of the offspring during UTEs testing the existence of distinct attachment types. The position of the points corresponds to the UMAP representation, while the colours attributed correspond to the spectral cluster analysis. NN, number of nearest neighbors. The axis of both plots represents a dimension reduction of the original data to two dimensions. Bottom: interindividual variation of offspring reactions during UTEs (results of the PCA). The colours represent the attachment types attributed based on the UMAP method and the cluster analysis. The bigger points represent the average loading for each attachment type category. The arrows represent the different dimensions (behavioural reactions) that have been reduced into two dimensions (top). Attachment type 1 corresponds to individuals not showing specific behaviour more often than expected for their age during UTEs; attachment type 2 corresponds to individuals not reacting during a threat or climbing, approaching another individual or running away without orienting towards the mother to seek safety more than expected for their age; and attachment type 3 corresponds to individuals approaching and looking towards their mother more than expected for their age. The sample size is *n* = 18 individuals.

Given that we found strong individual differences in key attachment-related variables (Fig. 2b), we explored whether individuals displayed consistent behavioural patterns regardless of age and other social factors. Using the unsupervised Uniform Manifold Approximation and Projection (UMAP) method, we identified three clusters without predefined numbers. A supervised cluster analysis with three set clusters produced similar results, consistently grouping the same individuals (Fig. 3a).

The principal component analysis (PCA) results indicated which behaviours drove each cluster (Table 2 and Fig. 3b). The first PCA component explained 65% of the variance, with ‘approaching the mother’ contributing the most (eigenvalue of 2.01). Adding component 2 explained 79% of the variance (eigenvalue of 0.43), driven by ‘approaching other individuals’. Including component 3 explained 90% (eigenvalue of 0.34). Each remaining component explained less than 5%. Attachment type 1 was attributed to individuals near the PCA centre, following model predictions. Attachment type 2 individuals relied less on their mothers than expected, while attachment type 3 individuals approached their mothers more than predicted for their age (Fig. 3b).

**Table 2:** Contribution of each behaviour of the offspring reaction during UTEs to the three first components of the PCA

	Component 1	Component 2	Component 3
Eigenvalue	**2.01**	0.43	0.34
Cumulative variance per cent	**65.30**	**79.09**	90.20
Not reacting	−0.36	−0.24	−0.26
Looking towards the mother	0.20	−0.06	0.01
Approaching the mother	**0.87**	0.16	−0.00
Approaching another individual	−0.07	0.70	−0.67
Running away	−0.12	0.02	−0.11
Climbing up a tree	−0.23	0.61	0.52
Whimpering	0.02	0.01	−0.02
Screaming	−0.03	−0.11	−0.04
Attacking or arm waving	−0.08	0.20	0.45

The results of the PCA include eigenvalues, cumulative variance per cent and scores of variables for each component. The numbers in bold represent the variable that contributes the most negatively or positively to each component. The sample size is *n* = 30 individuals across 550 independent events.

#### Mother–offspring proximity during social exploration

**Fig. 4:**
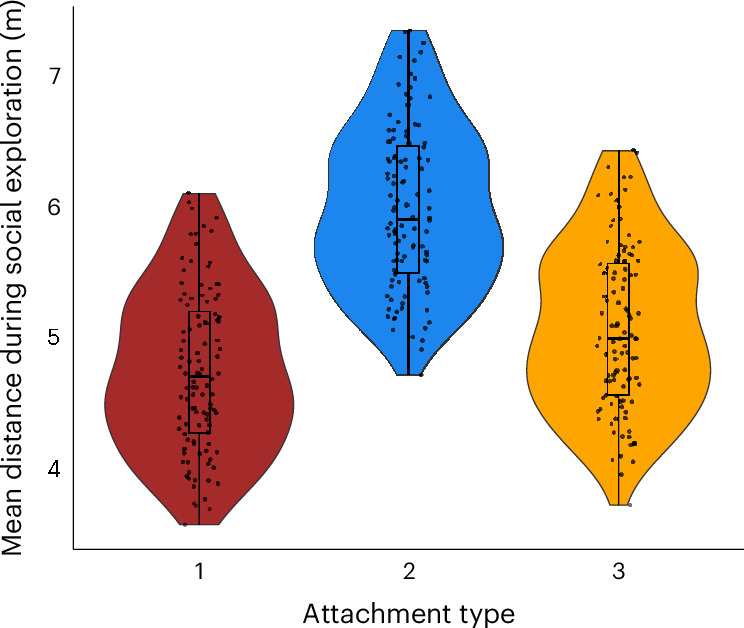
Mother–offspring proximity during social exploration with attachment type (model 3). Each dot corresponds to a focal follow on a given offspring. The *y* axis represents the model estimate of the mean distance between the offspring and its mother per focal. The box plots show the median and the interquartile range from the 25th to the 75th percentile of the posterior distribution. The whiskers indicate the range of data within 1.5 times the interquartile range from the lower and upper quartiles. Based on our prior analyses (UMAP, cluster analysis, PCA and model 2), attachment type 1 corresponds to secure attachment, attachment type 2 aligns with insecure-avoidant attachment and attachment type 3 corresponds to insecure-resistant attachment. The star represents an 89% CI excluding zero, indicating a credible effect. The sample size is *n* = 18 individuals across 260 independent events.

To validate the attribution of attachment types to each individual based on their behavioural reactions during UTEs, we investigated whether there were differences in mother–offspring distances during social exploration across focal samples depending on attachment type. The exploratory events were not related to the UTE. We modelled the mean distance between offspring and mother per focal day predicted by the attachment type and other control variables (for example, sex and age) (model 3), with the results presented in Table 3. The conditional *R*^2^ was 0.279. We showed an effect of attachment type with 89% of CIs. Indeed, offspring with attachment type 2 engaged in social exploration further away from their mother than offspring with attachment type 1 (Fig. 4) Control variables produced no consistent difference in mother–offspring proximity (all 89% CI overlapped 0; Table 3). Only daily mean party size showed a notable trend, suggesting that larger social gatherings were associated with offspring engaging in social exploration further away from their mother.

**Table 3:** Attachment type predicting mother–offspring proximity during social exploration: the result of model 3

	Estimate	Estimate error	95% CI	89% CI
Intercept	5.11	0.92	(3.34 to 6.95)	(3.65 to 6.60)
AT–AT3	−0.34	0.63	(−1.59 to 0.91)	(−1.34 to 0.66)
AT2–AT3	0.93	0.70	(−0.47 to 2.26)	(−0.20 to 2.04)
AT2–AT1	−1.27	0.78	(−2.81 to 0.29)	(−2.49 to −0.04)
Offspring age	0.31	0.50	(−0.66 to 1.32)	(−0.48 to 1.11)
Mother age	0.34	0.43	(−0.54 to 1.16)	(−0.37 to 1.00)
Percentage swelling	0.05	0.25	(−0.46 to 0.53)	(−0.36 to 0.45)
Sex	−0.25	0.72	(−1.62 to 1.16)	(−1.38 to 0.90)
Group South	0.74	0.69	(−0.62 to 2.05)	(−0.37 to 1.83)
Group East	−0.45	0.68	(−1.83 to 0.92)	(−1.54 to 0.63)
Mother rank	0.01	0.40	(−0.76 to 0.82)	(−0.62 to 0.65)
Party size	0.45	0.29	(−0.12 to 1.01)	(−0.01 to 0.91)
AT1: offspring age–AT3: offspring age	0.27	0.60	(−0.91 to 1.44)	(−0.68 to 1.21)
AT2: offspring age–AT3: offspring age	0.50	0.79	(−1.10 to 1.99)	(−0.81 to 1.73)
AT2: offspring age–AT3: offspring age	−0.66	0.9	(−2.45 to 1.13)	(−2.09 to 0.80)

AT1, AT2 and AT3 correspond to attachment type 1, attachment type 2 and attachment type 3. The 95% and 89% CIs are the CIs at their respective confidence levels. The reference levels for each predictor are female for ‘Sex’, and North for ‘Group’. The numbers in bold represent CIs excluding 0. The sample size is *n* = 18 individuals across 260 independent events.

#### Comfort after a threatening event

**Fig. 5:**
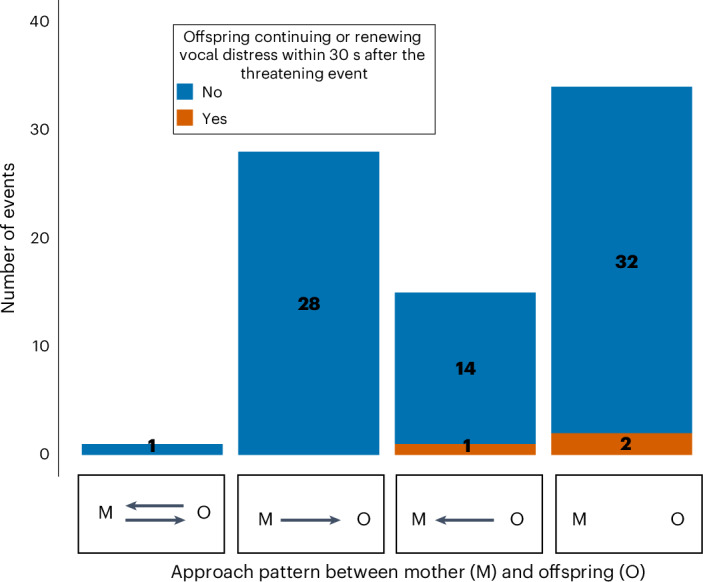
Assessing the validity of insecure-resistant attachment pattern: mother–offspring approaches following offspring vocal distress and subsequent response within 30 s. We assessed comfort seeking (offspring approaches mother) and comfort giving (mother approaches or touches offspring), represented by the arrows, or self-comfort (no new behaviour sign of distress of the offspring within 30 s after the threatening event without approach from either mother or offspring, no arrow). Blue represents offspring stopping or not renewing vocal distress within 30 s after the threatening event and orange represents offspring continuing or renewing vocal distress within 30 s after the threatening event suggesting no immediate comforting. The sample size is *n* = 18 individuals across 78 independent events.

Finally, we investigated whether the offspring continued being distressed even after their mother had comforted them, to distinguish between secure and insecure-resistant attachment (Fig. 5). During the events when the mother approached or physically made contact, the offspring never continued or renewed vocal distress. In only 3 of 78 events did offspring continue or renew vocal distress within 30 s of experiencing a threat. During these three events, the mother did not approach or make physical contact with the offspring, nor did she react to further distress.

Next, we analysed the variation in the duration spent by offspring exploring their environment in both social and non-social contexts per day in relation to attachment type. Our findings indicated that there was no discernible effect of attachment type on the duration spent by offspring exploring their environment in either social or non-social contexts (Supplementary Table 3). Finally, we investigated whether attachment types could predict the latency for offspring to resume exploration after experiencing a UTE or a threat during a play bout. There was no difference between attachment types 1 and 3 in latency time (Supplementary Table 4).

The results of these additional analyses are explained in Supplementary Text 1.

## Discussion

Our dataset of wild chimpanzees showed no evidence of disorganized attachment. This differs from what has been observed in humans and captive chimpanzees^33,34^. Instead, the responses of immature, wild chimpanzees during threatening situations revealed organized attachment patterns comparable to the secure (attachment type 1) and insecure-avoidant (attachment type 2) attachments found in humans^5^. Offspring who rarely approached their mother during a UTE explored socially from a greater distance than those who sought maternal proximity.

However, there was no strong support for a difference in mother–offspring proximity during social exploration between individuals with a secure-like attachment type and those with an insecure resistant-like attachment type (type 3). Additionally, there was no evidence of young chimpanzees continuing vocal distress after receiving comfort, indicating the absence of an insecure-resistant attachment axis. Finally, offspring did not exhibit aggressive, contradictory or abnormal behaviours typically associated with disorganized attachment.

Our findings suggest that certain characteristics of organized attachment have a deep evolutionary history. Meanwhile, the lack of disorganized attachment features in wild chimpanzees lends support to the assertions that this phenotype is indeed maladaptive.

### Variations of behavioural reactions to threat across age

The results revealed age-dependent responses to threats. As expected, offspring became more independent with age, relying less on their mother and acquiring skills to navigate and respond to potentially threatening situations, such as running away or climbing trees for self-protection (Fig. 2a). During weaning (at approximately 4 years old^28^), offspring were less likely to react to threatening events. We speculate that maternal availability may be limited following the birth of a new offspring, inhibiting approaches from both mother and weaned offspring before the latter has fully developed more independent strategies (for example, climbing up a tree or running away). Across animals, maternal reproductive strategies are finely balanced: mothers gain by reproducing with shorter inter-birth intervals but only if the older offspring survives^40,41^. Hence, when younger siblings are born, older siblings who show inappropriately low reactivity to threats may still be reaching suitable levels of independence. These findings support the notion that the weaning period, precipitating greater social and nutritional independence, plays a crucial role in ontogeny, being linked with the emergence of complex behaviours such as call combinations^42^, social grooming and tool use^43^.

### No evidence of disorganized attachment

The 50 subjects in this study did not show behavioural patterns corresponding to the disorganized attachment type found in humans and in captive chimpanzees^33^. In humans, disorganized attachment is found in 23.5% of children tested across cultures using the SSP^6^ and in 54% of institutionalized children^44^. Children and other primates with disorganized attachment demonstrate aggressive behaviour towards their mother, caregiver and peers, and exhibit abnormal or conflicted, confused or apprehensive behaviour. The Taï chimpanzees observed here did not show any of these behaviours. Given that aggressive or abusive behaviours from mothers towards offspring are considered a common cause associated with disorganized attachment^14^, we looked at the aggression rate per hour from mothers to their offspring. Events, when the mother showed aggressive behaviours towards her offspring, were very rare (48 aggressions over 3,795 h of observation among 17 of 33 mothers). The mean rate of aggression per mother–offspring dyad was 0.008 per hour for non-contact aggression (maximum rate at 0.10 per hour; arm waving) and 0.003 for contact aggression per hour (maximum rate of 0.03 per hour; hitting). The low rate of contact aggression from mother to offspring in wild chimpanzees is also supported by other studies (Reddy and Sandel^45^: no contact aggression observed in 29 young males over 1,570 h and Sabbi et al.^46^: 85 aggressions observed towards 49 immature chimpanzees over at least 4,116 h (maximum 0.02 per hour)). As a comparison, female chimpanzees direct contact aggression towards other individuals 2.3 times more frequently than towards their offspring, and non-contact aggression 2.5 times more frequently towards other individuals than their offspring (extrapolated data from the dataset of Tkaczynski^47^). Altogether, this suggests that the disorganized attachment type is rare or absent in wild chimpanzees. If it is present at an early age, those infants probably do not survive. In the wild, 15% of chimpanzee infants die before the age of 1.5 years^48^. Our findings corroborate the hypothesis that the disorganized attachment type is not an adaptive survival strategy in the face of environmental constraints in a wild setting.

In contrast, for orphaned chimpanzees in captive settings, behaviours indicative of disorganized attachment have been reported at relatively high rates^33,34^. Specifically, 41% of chimpanzees receiving responsive care and 72% of those under standard care from keepers exhibited disorganized attachment with the favourite human caregiver shown by sequential or simultaneous displays of contradictory behaviours or misdirected attachment behaviour. Compared with wild settings, captive settings provide an environment in which threats to survival are low, such as no exposure to predators nor out-group lethal aggression, health is managed and food is provisioned to reduce competition. However, psycho-social risk factors may be higher due to historical issues such as some zoo chimpanzees, that are now mothers, may have been rescued from isolated- or peer-housed living in medical facilities. As such, captive chimpanzee social scenarios may show parallels with those of modern human societies, where survival is less dependent on socio-ecological factors and high survival rates probably facilitate greater variation in psycho-social phenomena.

Disorganized attachment can result from caregivers displaying frightening behaviours towards children^12,49^. Specifically, offspring who have experienced sustained/extreme aggressive or neglectful maternal care, are at substantially higher risk of developing extreme psychopathologies in adulthood^50^. In contrast, the maternal behaviour of chimpanzees observed in our study lacked evidence of the abusive behaviours observed in human contexts. In Taï chimpanzees, extreme rejecting or neglectful maternal behaviour is exceptionally rare. Only two instances of abandonment were observed across 85 cumulative observation years of three communities. In each case, and unusual in chimpanzees, the maternal grandmother was present (Wittig and Crockford, personal observations). In contrast, instances of inadequate maternal care in zoos leading to humans taking over offspring rearing occurred for 8 infants involving 19 mothers across less than 5 years^51^ and for 7 infants involving 23 mothers across 9 years^52^.

Orphaned captive chimpanzees might develop disorganized attachment since, even with excellent care, they generally lack both a 24 h and permanent attachment figure to offer nurturing and protection and typical early social interactions with a primary caregiver of the same species. Both may explain the high percentage of disorganized chimpanzees in the studies of Van Ijzendoorn and colleagues^33^ and Clay^34^. It has been suggested that attachment and maternal nurturing propensity are transmitted across generations (in humans: Van Ijzendoorn and Bakermans-Kranenburg^53^; in mammals: Rilling and Young^54^), implying that individuals raised by nurturing mothers are likely to demonstrate nurturing care to their own offspring, as well as conversely. Animal welfare in captivity has become a welcome priority in recent years as understanding of the long-term impact of rearing conditions has improved^55^. Consequently, mothers born and raised in captivity or adopted from medical facilities a generation (20–30 years) ago might not have received adequate care as offspring, potentially leading to poor mothering skills with a higher likelihood of abandoning their own offspring or demonstrating reduced sensitivity to their needs^56,57^.

### Evidence of organized attachment

We demonstrated that young wild chimpanzees show variation in their reliance on their mother during UTEs, even after controlling for their age. This finding, driven principally by approach behaviour towards mothers, implies that some individuals use their mothers more as a safe haven during a UTE than others. Therefore, like in humans, chimpanzee offspring could be considered to have different organized attachment types. Some individuals did not demonstrate age-appropriate reliance on their mother during UTEs (attachment type 2), fitting well the criteria for insecure-avoidant attachment or excessive reliance on the mother (attachment type 3), similar to the insecure-resistant attachment (Fig. 3). Others showed age-appropriate reliance on their mother (attachment type 1) akin to the secure attachment (Fig. 3). To better understand individual differences and the adaptability of organized attachment, it is now necessary to investigate maternal responsiveness and determine whether the developed attachment is related to the mother’s responsiveness.

An additional measure to distinguish between secure and insecure-resistant offspring’s behavioural reactions examined whether offspring showing distress during exposure to a threat are comforted after seeking comfort from their mother. In humans, children with insecure-resistant attachment persist in displaying distress even when the mother attempts to provide comfort. In our sample, offspring rarely continued or renewed vocal distress after a threatening event (Fig. 5). Rare instances where offspring continued to seek their mother were observed only when the mother did not offer comfort. This observation strongly suggests that in the wild, young chimpanzees are reliably comforted by their mothers if comfort is provided, challenging the notion of the existence of insecure-resistant attachment. The individuals initially considered as insecure resistant in our dataset could be secure offspring overreacting during UTEs (which is a subcategory of the secure attachment). This overreaction could be due to the offspring’s temperament, particularly their susceptibility to distress and anxiety, rather than being solely attributed to maternal factors^58,59^. In 32 out of 78 observed instances, there was no approach between mother and offspring, yet the distress subsided regardless. This behaviour might imply the use of self-comforting mechanisms or growing emotional independence. Furthermore, 14 out of 18 individuals exhibited this behaviour at least once. Notably, these instances often occurred during play bouts, indicating that the initial threat was mild and soon over, thus not requiring further comfort.

Furthermore, using the same focal data for analysis to determine the different attachment types (test B1) and incorporating new data from threat occurrences during play bouts, we examined (Supplementary Table 4) whether attachment types predicted the latency time for offspring to explore after a UTE or a threat during a play bout. This analysis revealed no strong differences, supporting the idea that attachment types 1 and 3 might demonstrate no functional difference. The findings presented in Table 3, which model the proximity between mother and offspring during social exploration unrelated to threatening events, support the notion of individual distinctions between offspring exhibiting attachment type 1 (secure-like) and attachment type 2 (insecure avoidant-like). Indeed, offspring with attachment type 2 explored further away from their mother in a social context than offspring with attachment type 1 (Fig. 4). Insecure avoidant-like individuals did not rely on their mother during UTEs, exploring further away from her, consistent with the idea that they did not consider her as a safe base. There was no difference between offspring with attachment type 1 (secure-like) and attachment type 3 (insecure resistant-like), again corroborating the idea that those individuals might all show degrees of ‘secure’ attachment. Contrary to our prediction that securely attached offspring (attachment type 1) would explore their environment further away from their mother than insecure-resistant offspring (attachment type 3), the results indicated that attachment type 1 offspring actually explored closer to their mother than attachment type 3 offspring, yet with no substantial differences (no effect at 89%).

Overall, these results align with the findings of Van Ijzendoorn and colleagues^33^, who identified diverse organized attachment types (secure, insecure avoidant and insecure resistant) in captive chimpanzees that resembled those observed in humans. However notably, we did not yield evidence supporting the presence of insecure-resistant attachment in wild chimpanzees, possibly due to our comparatively smaller sample size, that is 18 versus 46 individuals.

### Limitations

Given our small sample size for the assessment of chimpanzee attachment types, our sample may not have captured the full diversity of attachment in young chimpanzees. Nevertheless, we showed strong evidence for interindividual variability. Controlling for offspring age posed challenges due to the limited number of individuals, resulting in few subjects with identical ages. Nevertheless, we maintained confidence in the validity of our methodology by controlling for age differences, asserting that attachment types can still be assessed in older offspring within the range of 1.5–6 years. A strength of our approach was having repeated observations in natural situations across time, which probably increases the capacity to distinguish variation in attachment. This contrasts with the SSP in humans, which is typically conducted only once per child, out of their natural environment, in a safe place and notably only in certain cultures^5,6^. Additionally, mother-initiated approaches could limit direct comparability to human studies, where mothers are told to not react, but this is unavoidable in a naturalistic setting where mothers behave freely. Additionally, behaviour not only in threatening contexts but also in daily social life, such as mother–offspring distance, was predictable based on reactions to threatening events in wild immature chimpanzees, further highlighting parallels to organized attachment in humans. Future research is needed to explore how attachment type relates to social behaviour across the lifespan. Finally, one might question whether organized attachment in humans is comparable to that observed in chimpanzees. In humans, organized attachment is defined by a child’s use of a consistent and coherent strategy to seek comfort and support from their primary caregiver when distressed^60^. Our findings suggest that wild immature chimpanzees exhibit behavioural strategies similar to those seen in human infants, such as approaching or avoiding their mother when facing potential threats, which is a situation analogous to the SSP experiments. This convergence in behavioural patterns, despite differences in the nature of the stimuli (experimental versus naturally occurring), suggests a shared foundation in attachment strategies between chimpanzees and human infants. Additionally, behaviour not only in threatening contexts but also in daily social life, such as mother–offspring distance, was predictable based on reactions to threatening events in wild immature chimpanzees, further highlighting parallels to organized attachment in humans. Future research is needed to explore how attachment type relates to social behaviour across the lifespan.

## Conclusions

By examining the hugely influential psychological concept of attachment theory within an evolutionary framework, we were able to identify distinct mother–offspring attachment types in young wild western chimpanzees, similar to the secure and insecure-avoidant types found in humans, but no evidence of disorganized attachment. While this does not rule out the occurrence of disorganized attachment in wild chimpanzees, it suggests that if it occurs, it does so at much lower rates than is found in humans or peer-raised captive chimpanzees. Thus, these findings support the hypothesis that disorganized attachment may not be adaptive for offspring survival. Future research should investigate the impact of attachment types on offspring fitness, and the relation between maternal care and attachment types in other mammals. Our findings provide a crucial step towards understanding how adaptive attachment strategies emerge across species, as well as the key role attachment may play in social evolution. Shared attachment strategies across species may indicate a common evolutionary heritage, underscoring the ancient origins of sociality in primates. However, the relatively high prevalence of disorganized attachment in both human and captive chimpanzee populations is consistent with the hypothesis that rearing environments contribute substantially to its manifestation and persistence in certain contexts. This contrast between the lack of disorganized attachment in the wild setting of our study and the relatively high prevalence of disorganized attachment in captive environments indicates a need for further research to understand the underlying causal factors (for example, genetic influence and parental state of mind)^61^. Further comparative research in natural environments is beneficial for understanding mother–offspring attachment dynamics, which are crucial for primate social development and for other long-lived mammals with protracted development. Observing these dynamics in natural settings reveals how ecological pressures and social contexts shape attachment behaviours, offering insights that controlled environments often overlook.

## Methods

### Ethics statement

Our study was purely observational and non-invasive. Observers followed the strict hygiene protocol of the Taï Chimpanzee Project, adopted by the International Union for Conservation of Nature as the best practice guideline for wild ape studies^62^. Observers quarantined for 5 days before following the chimpanzees. Every day, observers disinfected their hands and boots and changed clothes before leaving and entering camps. In the forest, observers wore face masks and kept a minimum distance of 7 m between themselves and the chimpanzees, to avoid disease transmission from humans to chimpanzees and to avoid disturbing the natural behaviour of the observed individuals. The research protocol used here was approved by the ‘Ethikrat’ of the Max Planck Society on 04 August 2014 and by the ‘Directeur de Recherche en Côte d’Ivoire’ under the Permit TCP Wittig/008/MESRS/DGRI from 05 May 2021.

### Study site, subjects and data collection

E.R., O.N.-L., P.J.T. and research assistants collected behavioural data on three wild Western chimpanzee (*P.* *t.* *verus*) communities (North, South and East groups), habituated to human observers and located in the Taï National Park, Côte d’Ivoire^63^.

Behavioural focal sampling followed a standardized methodology, with two focal individuals observed daily for about 6 h each from approximately 6:30 to 12:30 and 12:30 to 18:30 (ref. ^64^). Cybertracker (v.3.389, v.3.440 and v.3.517) was used to record daily activities (resting, eating, walking and so on), social interactions (grooming, playing, affiliations, aggressions and solicitations), mother–offspring proximity and mother–offspring interactions. Subgroup composition and changes in behaviour were also continuously noted. Reliability of the coded behaviours and individual identities was high across datasets between coders (Cohen’s Kappa tests: *ĸ* = 0.72, 0.81, 0.70, 0.78, 0.80 and 0.64)^65^. Full ethogram details are available in the supporting information^66,67^ (Supplementary Table 1).

### Threatening events

To correspond with the SSP^68^, which involves a potential threat to the infant, we chose naturally occurring situations of undirected aggressions, or aggressions and threats not directed towards the offspring (excluding the presence of predators) for analysis (UTEs). In chimpanzees, third-party aggressions can result in redirected aggressions to nearby individuals^69^. Additionally, we included the following contexts of UTEs that typically alert the group: chimpanzee vocalizations outside of the group, alarm calls of other species and gunshots. We removed events when the offspring was already in contact with the mother and when the mother was included in the aggression to account for the mother’s availability for their offspring. To assess responses to vocal distress (tests A1 and B3 in Fig. 1), we added threats directed at the offspring that more regularly triggered vocal distress, including threats during play bouts and aggressions directed towards the offspring (DTEs). Behavioural reactions of both the mother and offspring were recorded during these potentially threatening situations (immediately, that is, within approximately 2 s).

### Statistical analyses

We performed the following analyses using R Studio (R version 4.2.2)^70^ and Spyder Python (version 3.9)^71^.

We first tested the existence of disorganized attachment by assessing whether distress during a UTE or DTE predicted approach behaviour between mother and offspring (test A1) and analysing aggression rates between mother and offspring (test A2). We then tested the existence of distinct organized attachment types by analysing offspring reactions to UTEs (controlling for age and other variables; test B1a in Supplementary Fig. 1), applying dimension reduction and cluster analysis to detect patterns (test B1b in Supplementary Fig. 1). Furthermore, we examined differences in the mean distance between mother and offspring during social exploration excluding threatening events (test B2) and evaluated the existence of insecure-resistant attachment by assessing the effectiveness of maternal comfort after a UTE or DTE (test B3). The rationale for the different steps of analyses and the respective data used are shown in Supplementary Fig. 1.

#### Testing the existence of disorganized attachment

##### Comfort-seeking during distress

**Model 1**

If wild immature chimpanzees have a disorganized attachment type, we predicted that vocal distress would not result in comfort seeking with their mother. We used all threatening events (UTEs and DTEs) and modelled whether the presence of offspring distress (whimpering and screaming) would cause offspring and mother to reduce their distance (seeking or offering comfort, respectively) using a Bayesian generalized linear mixed model (GLMM) with a Bernoulli error distribution. The control variables are explained in Supplementary Text 1. Each UTE or DTE constituted a data point, totalling 567 observations across 30 individuals (ages 0–10 years). We extracted the estimates of the random slope for whimpering or screaming occurrences associated with the identity of the individuals to evaluate how strongly vocal distress predicted the probability of the mother–offspring approach for each dyad.

##### Aggression between mother and offspring

Disorganized attachment can be identified in situations where the offspring exhibits fear towards the caregiver. If an offspring consistently displays aggression towards their mother in a fearful context, it suggests a disorganized attachment, particularly if the mother exhibits aggressive behaviour at a higher rate than other mothers towards their offspring. Therefore, to identify whether some individuals presented disorganized attachment, we investigated how often there was aggression observed between mother and offspring. We counted the number of occurrences of both contact and non-contact aggressions between mother and offspring in 50 mother–offspring dyads (ages 0–10 years). Contact aggressions were described as hitting, pushing away, and pulling an individual. Non-contact aggressions were described as hunching, doing an arm wave, charging or chasing an individual (see ethogram in Supplementary Table 2).

#### Existence of different organized attachment types

##### Reactions of offspring during threatening events

To investigate the existence of different types of attachment in immature chimpanzees, we explored the variability between individuals in behavioural reactions during threatening events. Given that our dataset contained individuals from 0 to 10 years old and that we expected immature wild chimpanzees to rely less on their mothers with age, we first ran a model (model 2) controlled for age and other variables. We then used dimension reduction approaches and cluster analysis to see whether within and between individual correlations in these behavioural responses existed.

**Model 2**

We expected offspring to react differently to a threat depending on age, with younger individuals relying more on their mothers. Therefore, to control for the variation of age across individuals, we used Bayesian models with a Bernoulli error distribution. Each UTE constituted a data point, totalling 550 observations across 30 individuals of data between 2016 and 2023 (ages 0–10 years), and the response variable was again a binary value of the occurrence of the behavioural reaction during an event. Individuals could show multiple reactions during the same UTE.

We ran a separate Bayesian GLMM for each of the nine behaviours (approaching the mother, looking towards the mother, whimpering, screaming, running away from the threat, climbing up a tree, attacking, approaching another individual or not reacting). For visual representation (Fig. 2), we ran a similar model with all the behavioural reactions into a matrix (model 2). We used the offspring age in months on the day of the event as a test predictor. The control variables are explained in Supplementary Text 1. The random intercepts for individuals were extracted, representing deviations from the population-level intercept (average behavioural response), accounting for age and other fixed factors. This captured individual variability not explained by the fixed effects. Positive values meant that individuals showed the given behaviour more than expected for their age and other fixed effects, and less than expected for negative values.

Owing to older offspring (>6 years old) exhibiting fewer mother-dependent behaviours such as approaching and looking towards the mother and displaying more independent behaviours such as climbing up a tree and running away from threats (Fig. 2), we excluded them from subsequent analyses. Young individuals (<1.5 years old) were omitted from further analysis, similar to the practice of excluding very young human infants in the SSP owing to their consistent proximity to their mother, which posed challenges in assessing variations in mother-directed behaviours. This resulted in 18 dyads (*M* = 3.7 years, range of 1.5–6 years, s.d. 2.4).

We investigated whether individuals exhibited consistent behavioural patterns regardless of their age and the other control variables. We used dimension reduction techniques (UMAP and PCA) and a spectral cluster analysis. We aimed to discern whether individuals aged between 1.5 and 6 years displayed distinct behavioural reactions across 309 UTEs and could be categorized into clusters. We applied these techniques to the deviation estimates extracted from model 2 on 18 individuals (ages 1.5–6 years). More details about these methods are described in Supplementary Text 1.

##### Mother–offspring proximity during social exploration

**Model 3**

To investigate whether attachment type predicted mother–offspring proximity during social exploration, we analysed the average distance between mothers and their youngest offspring during social play (excluding the mother as a partner). Attachment type was determined using dimension reduction techniques and cluster analysis previously explained (test B1). We applied a Bayesian GLMM with a Gaussian error distribution for this analysis. Each date of data collection per individual constituted a data point, totalling 260 observations across 18 individuals (ages 1.5–6 years).

The mother–offspring distances were categorized and a value corresponding to the mean distance was attributed as follows: contact (value of 0), less than 1 m (value of 0.5), between 1 m and 5 m (value of 3), between 5 m and 10 m (value of 7.5) and more than 10 m (value of 15). The mean distance during social exploration per date per individual was calculated as the sum of every distance multiplied by the time spent at that distance divided by the total observation time of social exploration of that date (sum(value.distance × (duration.distance/duration.total.date))). We used attachment type in interaction with the age of the offspring in months as a test predictor, since we expected that the impact of attachment type on mother–offspring proximity during social exploration would differ across different stages of offspring development. Justifications for including the control variables are given in Supplementary Text 1.

Additionally, we modelled the percentage of time spent by the offspring exploring their environment in non-social (model 4) and social contexts (model 5) with the attachment type (model explanation in Supplementary Text 1 and results in Supplementary Table 3).

##### Comfort after a threatening event

To disentangle secure and insecure-resistant attachments, we investigated whether offspring continued being distressed even after their mother had comforted them. We took all the UTEs and frightening events during play bouts that elicited offspring whimpering or screaming and plotted whether they continued or renewed vocal distress (whimpering or screaming) after the distance between mother and offspring was reduced between the moment of the threat and until 30 s after (Fig. 5). There were 78 observations across 18 individuals (ages 1.5–6 years).

Additionally, we performed a survival analysis (model 6) to investigate the existence of insecure-resistant attachment by looking at the latency time between a threatening event and exploratory behaviour (Supplementary Text 1 and Supplementary Table 4). On the basis of the human literature, we hypothesized that insecure-resistant offspring would take longer to explore their environment after a threat compared with secure and insecure-avoidant offspring.

We ran all models in R 4.2.2 (ref. ^70^) using the function ‘brm’ from the package ‘brms’^72^. All continuous values were scaled to a mean of 0 and a standard deviation of 1. We tested for collinearity issues by quantifying variance inflation factors for our predictor variables using the function ‘vif’ from the package ‘car’^73^. We ran 2,000 iterations (1,000 for ‘warm-up’) on 12 chains. We used weakly regularizing priors for the fixed effects (normal (0,1)) and the priors given by default by the function ‘get_prior’ of the package ‘brms’ for the random effects (that is, Student’s *t* (3, 0, 2.5) for the random intercepts and slopes). We then extracted the 95% and 89% CIs for each fixed effect from the posterior distribution of the model. Sampling diagnostics (*R*-hat of 1 for all predictors) and trace plots confirmed chain convergence for all models. Effective sample sizes (all >3,800) confirmed no issues with autocorrelation of sampling for all models. All variance inflation factors were below 5 confirming non-collinearity between variables. All posterior predictive checks for each model were satisfactory (Supplementary Figs. 5 and 6).

### Reporting summary

Further information on research design is available in the Nature Portfolio Reporting Summary linked to this article.

## Supplementary information

Supplementary Information Supplementary information.

Reporting Summary

## Data Availability

All data for the analyses supporting the findings of this study are available via GitHub at https://github.com/eleonorerolland/Attachment_types_chimpanzees.

## Code availability

All codes for the analyses supporting the findings of this study are available via GitHub at https://github.com/eleonorerolland/Attachment_types_chimpanzees.
