# Publisher Correction: Evidence of organized but not disorganized attachment in wild Western chimpanzee offspring (*Pan troglodytes verus*)

**DOI:** 10.1038/s41562-025-02263-w

**Published:** 2025-06-23

**Authors:** Eléonore Rolland, Oscar Nodé-Langlois, Patrick J. Tkaczynski, Cédric Girard-Buttoz, Holly Rayson, Catherine Crockford, Roman M. Wittig

**Affiliations:** 1 Ape Social Mind Lab, Institut des Sciences Cognitives Marc Jeannerod, University of Lyon (CNRS UMR 5229), Bron, France; 2 Department of Human Behavior, Ecology and Culture, Max Planck Institute for Evolutionary Anthropology, Leipzig, Germany; 3 Tai Chimpanzee Project, Centre Suisse de Recherches Scientifiques en Côte d’Ivoire, Cote d’Ivoire, Abidjan, Côte d’Ivoire; 4 Université Claude Bernard Lyon 1, Villeurbanne, France; 5 Research Centre for Evolutionary Anthropology and Paleoecology, School of Biological and Environmental Sciences, Liverpool John Moores University, Liverpool, UK; 6 ENES Bioacoustics Research Laboratory, Centre de Recherche en Neurosciences de Lyon, University of Saint-Etienne (CNRS, Inserm), Saint-Etienne, France; 7 Institut des Sciences Cognitives Marc Jeannerod, University of Lyon 1 (CNRS UMR 5229), Bron, France

**Keywords:** Animal behaviour, Human behaviour, Social anthropology

Correction to: *Nature Human Behaviour* 10.1038/s41562-025-02176-8, published online 12 May 2025.

In the version of the article initially published, the *x* axis of the top graph in Fig. 2 showed scaled age and has now been corrected to show age in months in the HTML and PDF versions of the article.

