# Supplementary material for: Evidence of organized but not disorganized attachment in wild Western chimpanzee offspring (Pan troglodytes verus)
Source: Nat Hum Behav. 2025 May 12;9(8):1571–82. doi: 10.1038/s41562-025-02176-8 (PMC12367522; doi:10.1038/s41562-025-02176-8)
Supplement: Supplementary file 1 — Supplementary information. [file 41562_2025_2176_MOESM1_ESM.pdf]

nature human behaviour

Supplementary information https://doi.org/10.1038/s41562-025-02176-8

Evidence of organized but not disorganized attachment in wild Western chimpanzee offspring (*Pan troglodytes verus*)

In the format provided by the authors and unedited

Table S1: Ethogram of behaviors collected on wild western chimpanzees

Category	Behavior	Description
**Activity**	Walk	Walking by itself on the ground for more than 30s. Does not include foraging behavior.
**Activity**	Rest	Lying, sitting, standing or sleeping, not involved in other activities.
**Activity**	Climb	Climbing a tree or between branches, not in a playing context, more details are given if the individual climbs to the ground, in the tree or between branches.
**Activity**	Eat	Removing a food item (leaf, fruit, mushroom, etc.) from the substrate, processing, putting into mouth, biting, chewing and swallowing it.
**Activity**	Food manipulation	Taking in its hand a food item (fruit, nut, etc.) and manipulating it. This behavior excludes eating.
**Activity**	Climb carried	Carried by another individual climbing a tree (to the ground, between branches or to the tree).
**Activity**	Walk carried	Carried by another individual walking on the ground.
**Activity**	Play	Playing alone or with partners. Engaging in a social play interaction includes the following behaviors: climbing, running, chasing, swinging, pirouette, somersault, dragging object, tickling, biting, grabbing and so on. Social play can be associated with a laugh vocalization (panting). Solitary playing includes the following behaviors: pirouette, climbing, swinging, jumping, object manipulation such as throwing or dragging, etc.
**Activity**	Observe	Staring at another individual or an object for more than 5 seconds, from more than 50 centimeters, is different from peering.
**Activity**	Explore	Displacement in a limited area in order to forage, encounter with individuals or other activity related to the discovery of the environment
**Activity**	Nest	Constructing a platform for sleep or rest by bending branches over each other.
**Activity**	Nest carried	Carried by another individual making a nest.
**Activity**	Suckle	Sucking from her nipples.
**Affiliation**	Embrace	Two individuals facing each other and each putting one or both arms around the other.
**Affiliation**	Kiss	Pressing his or her lips against, or briefly laid against, the body, face, or limbs of another.

**Affiliation**	Touch	Reaching out with a hand (or occasionally foot) and touching another, with fingers (or toes), by laying the whole palmar surface of the hand on the other's body.
**Affiliation**	Mount	Embracing another with both arms from behind.
**Affiliation**	Sniffing	Putting nose close to another individual.
**Affiliation**	Finger mouth	Inserting one or more fingers into the mouth of another individual.
**Aggression (contact)**	Hit	Bringing down an arm from above and striking the partner with the fist.
**Aggression (contact)**	Push away	Shove away from another individual with one or both hands.
**Aggression (contact)**	Pull	Grasp and tug at another individual by flexing arms.
**Aggression (non-contact)**	Charge	Running directed toward another individual.
**Aggression (non-contact)**	Chase	Running after a fleeing individual, in order to grab it in aggression.
**Aggression (non-contact)**	Waving an arm	Raising and lowering one or both arms in a rhythmic or waving motion.
**Aggression (non-contact)**	Hunch	Hunching of shoulders.
**Grooming**	Grooming (actor or receiver)	Using both hands, pushing the hair back with the thumb or index finger of one hand and holding it back while picking at the exposed skin with the nail of the thumb or index finger of the other. Can also use one hand, parting the hair in the same way and holding it back with the lower lip. Grooming may occur in bipedal, quadrupedal, sitting, or lying posture. Function of grooming includes appeasement, reassurance and reconciliation in addition to elimination of ectoparasites.
**Sharing**	Food sharing	Allowing another individual to take food that is under the control of the owner (for example, in the mouth or hand, on the lap, or in proximity). Meat is the most common food to be shared among adults. Food sharing is most common between mothers and infants. Food sharing can be active or passive.
**Solicitation (response of partner)**	Vocalization	Emitting a sound part of the Vocalization category.
**Solicitation (response of partner)**	Grooming	See category Grooming.
**Solicitation (response of partner)**	Carrying	Partner carries the individual.

**Solicitation (response of partner)**	Affiliate	See category Affiliation.
**Solicitation (response of partner)**	Waiting	Stopping movement or activity.
**Solicitation (response of partner)**	Ignoring	Not responding to stimuli.
**Solicitation (response of partner)**	Aggressing	See category Aggression.
**Solicitation (response of partner)**	Nursing	Allowing the focal individual to suck from her nipples. See Suckle in Activity.
**Solicitation (response of partner)**	Playing	See Play in category Activity.
**Solicitation (response of partner)**	Sharing	See category Sharing.
**Solicitation (response of partner)**	Support	Intervening in the situation where the focal individual is in threat.
**Solicitation (response of partner)**	Avoiding	Moving away from the focal individual, either by turning back to him or her, or by displacement.
**Solicitation (way of solicitation)**	Gesture	Making movements with its body. This can include begging with hand by stretching hand to the possessor's hand, mouth or food or begging with mouth food by putting lips to the lips or hand of feeding possessors. Stamp in invitation described as a stamp on the ground with one foot while watching potential playmate, in invitation to social play or alloparental care (may be bipedal or quadrupedal).
**Solicitation (way of solicitation)**	Peering	Looking intently into another individual's face from a minimum of 50 centimeters. Peering does not usually elicit food sharing.
**Solicitation (way of solicitation)**	Contact	Touching another individual.
**Solicitation (way of solicitation)**	Whimper	See Whimper in Threat.

**Solicitation (way of solicitation)**	Temper tantrum	Screaming loudly and may leap up, fling arms above his/her head and then slap them onto the ground or beat the ground with his hands. The individual may rush off tumbling over and over, still screaming. The screaming often results in glottal cramps. Typically occurs among infants during weaning conflict, when mother rejects sucking, food sharing, transport, grooming and soon, however, even adults sometimes show in response to rejection by dominant individuals.
**Solicitation (way of solicitation)**	Leaf clip	Pulling a leaf repeatedly between lips or teeth with one hand, producing a conspicuous sound that attracts attention.
**Threat**	Approach mother	Moving towards the mother
**Threat**	Encounter with mother	The focal individual and the mother move towards each other ending with contact mother-offspring.
**Threat**	Look towards mother	Glancing at the mother.
**Threat**	Approach other	Moving towards another individual.
**Threat**	Encounter other	The focal individual and another individual move towards each other ending with contact other-offspring.
**Threat**	Climb	See Climb in Activity.
**Threat**	Run away	Moving away from the threat and not moving towards the mother.
**Threat**	Scream	See Scream in Vocalization.
**Threat**	Whimper	See Whimper in Vocalization.
**Threat**	No reaction	Not reacting to the situation according to any of the behaviors described in the category Threat.
**Threat**	Look towards focal	Glancing at the focal individual.
**Threat**	Approach focal	Approaching the focal individual.
**Threat**	Grab focal	Grabbing and carrying the focal individual.
**Vocalization**	Bark	Loud, sharp sounds, usually given in long sequences with much variation in pitch, wouah like sound, usually in a context of threat, the bark is considered as an alarm call.
**Vocalization**	Scream	High pitched and loud sound, almost always given in a series. Usually heard in contexts of aggression and general social excitement by highly stressed, fearful, frustrated, or excited individuals.
**Vocalization**	Grunt	Grunting sound with closed or open mouth.

**Vocalization**	Pant	Audible inhalations.
**Vocalization**	Intense greeting	Sequence of fast approach, eye contact, bobbing and intense pant-grunting.
**Vocalization**	Hoo	A single syllable soft whimper. A single hoo may be uttered several times in succession. It is the typical sound given by an infant which cannot reach the nipple and wants to reestablish contact with the mother and so on.
**Vocalization**	Pant hoot	Series of alternating pants and hoots with increasing volume that usually ends in a climax scream.
**Vocalization**	Greeting hoo	Repeated and intense series of hoos.
**Vocalization**	Pant grunt	Pants while inhaling and grunts with an open mouth while exhaling.
**Vocalization**	Whimper	Whole series of soft, low pitched sounds, rising and falling in pitch, which may lead to crying and screaming.
**Vocalization**	Rale	Vomiting like sound.
**Vocalization**	Tambourine	Hitting the buttress of a tree with hands and/or feet. Occasionally as part of a series of behaviors starting with a warm up (rocking and/or leaf clipping in front of the tree), pant-hooting, drumming and climax scream.

Table S2: Individuals and assigned attachment types

Individual	***Group***	***Sex***	***Median age (in months) during data collection***	***Median age (in years) during data collection***	***Mother***	***Attachment type***
SM1	South	M	23	1.9	SF31	Secure-like
SF2	South	F	66	5.5	SF31	-
SF3	South	F	8	0.7	SF32	-
SM4	South	M	14	1.2	SF33	-
EM5	East	M	37	3.1	EF34	Secure-like
SF6	South	F	58	4.8	SF35	Insecure-avoidant-like
EF7	East	F	25	2.1	EF36	Secure-like
SM8	South	M	25	2.1	SF37	Insecure-resistant-like

EM9	East	M	61	5.1	EF38	Insecure-resistant-like
EM10	East	M	62	5.2	EF38	Insecure-resistant-like
SM11	South	M	37 and 89	3.1 and 7.4	SF39	-
SF12	South	F	76	6.3	SF32	-
SF13	South	F	67	5.6	SF33	-
SF14	South	F	51 and 101	4.3 and 8.4	SF33	-
NM15	North	M	25	2	NF40	Insecure-resistant-like
NM16	North	M	74	6.1	NF41	Secure-like
NM17	North	M	37 and 90	3.1 and 7.5	NF40	-
NF18	North	F	60 and 120	5 and 10	NF41	-
SM19	South	M	69	5.8	SF42	Secure-like
EM20	East	M	60	5	EF43	Insecure-avoidant-like
EF21	East	F	55 and 110	4.6 and 9.2	EF43	-
SM22	South	M	41	3.4	SF44	Secure-like
NF23	North	F	28	2.3	NF45	Secure-like
SM24	South	M	44 and 98	3.7 and 8.2	SF44	-
SM25	South	M	50	4.2	SF46	Insecure-avoidant-like
SM26	South	M	40	3.3	SF47	Insecure-avoidant-like
SF27	South	F	38	3.1	SF48	Secure-like
SM28	South	M	56	4.6	SF49	Insecure-avoidant-like
SF29	South	F	51 and 101	4.3 and 8.4	SF49	-
EM30	East	M	55	4.6	EF50	Insecure-avoidant-like
SF51	South	F	52	4.3	SF31	-
EF52	East	F	101	8.4	EF71	-
EM53	East	M	97	8.1	EF72	-

EM54	East	M	55	4.6	EF36	-
SM55	South	M	61	5.1	SF73	-
EM56	East	M	69	5.8	EF38	-
EF57	East	F	105	8.8	EF75	-
NF58	North	F	68	5.7	NF76	-
SF59	South	F	70	5.8	SF74	-
NF60	North	F	50	4.2	NF77	-
EM61	East	M	122	10.2	EF43	-
NM62	North	M	85	7.1	NF45	-
EF63	East	F	64	5.3	EF78	-
NF64	North	F	70	5.9	NF80	-
SM65	South	M	80	6.7	SF79	-
SF66	South	F	72	6	SF81	-
SF67	South	F	124	10.3	SF81	-
SM68	South	M	47	3.9	SF47	-
EM69	East	M	96	8	EF82	-
EM70	East	M	98	8.2	EF50	-

For the individuals having two numbers in the age column, the values correspond to data collected during two distinct periods: 2016-2018 and 2021-2023, respectively.

Figure S1: Diagram of the methodological approach for the analyses

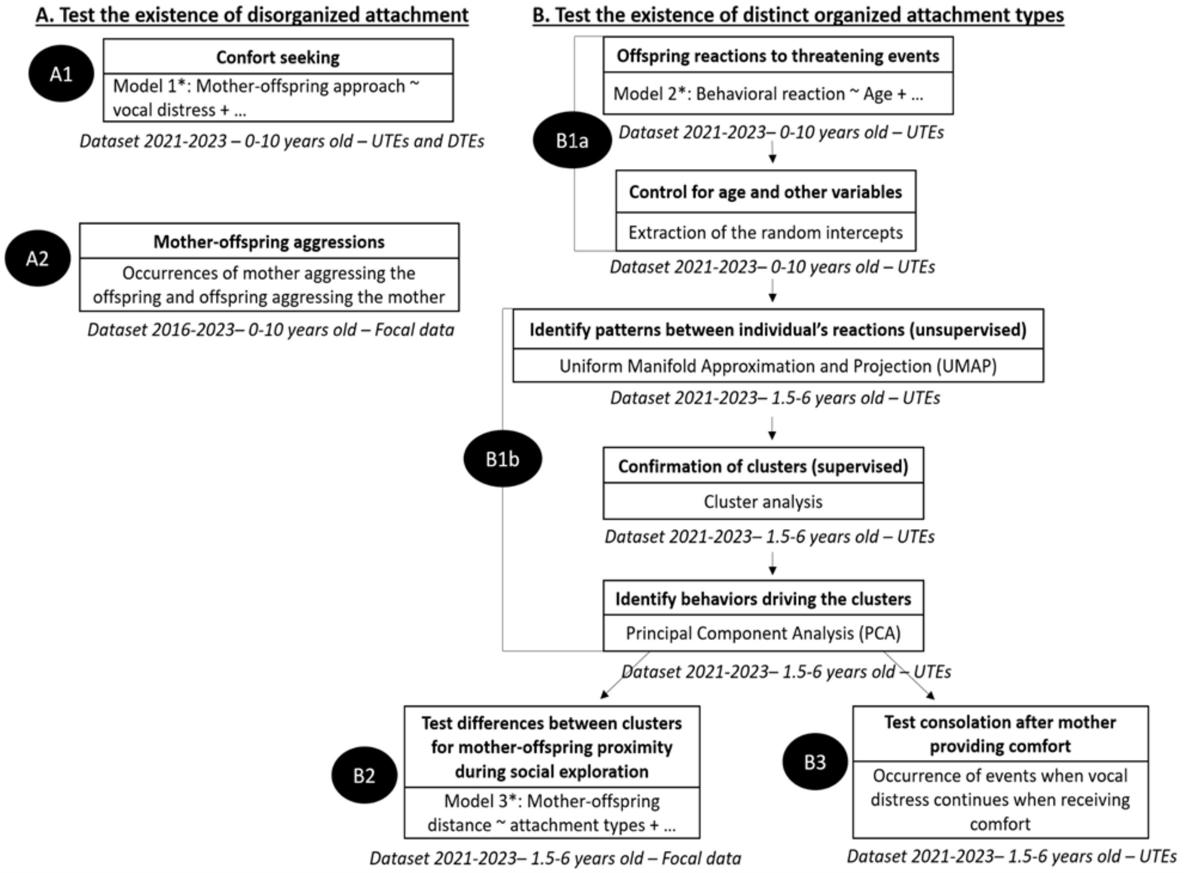

Figure S1. The letters with numbers correspond to each analysis conducted referring to the titles in the sections Material and methods, and Results, and the letters in **Figure 1**. Comfort-seeking during distress, A2. Aggression between mother and offspring, B1. Behavioral reactions of offspring during threatening events, B2. Mother-offspring proximity during social exploration, B3. Receiving comfort and being comforted after a threatening event. UTE refers to undirected threatening events and DTE refers to directed threatening events. Focal data: data collected during daily focal follows excluding the UTEs and DTEs. *Bayesian mixed models.

Text S1: Supplementary methods and results

Control variables for the different models and additional analyses

Control variables

Model 1. We included the following fixed effects based on their established impact on the social behavior of mothers and offspring: (1) offspring age[74], (2) offspring sex[75], (3) party size[39,76], and (4) group identity[77].

Model 2. We included the following fixed effects: (1) offspring sex[75], (2) presence of an older sibling[78], and (3) party size[39,76]. We coded the presence of older siblings in the party composition during the UTE as 1, and as 0 if no maternal sibling was present or if the individual did not have maternal siblings.

Model 3. We included the following control variables since they might influence the proximity of a mother to her youngest offspring: (1) the mother's age in years[79], (2) offspring sex[75], (3) party size[39,76], (4) rank of the mother[79], (5) percentage of time spent per day with at least one female with a full sexual swelling[80] and (6) group identity[77]. We controlled for the dominance rank of mothers because offspring with low-ranking mothers might be more susceptible to aggression, which consequently affects their distress levels. To calculate dominance ranks we used unidirectional submissive pant grunt vocalizations[81]. We applied a likelihood-based adaptation of the Elo rating approach[82,83,84] using the long-term data of the Taï Chimpanzee Project. Ordinal ranks were standardized daily between 0 and 1, with 1 being the highest rank. Since the presence of females with a full sexual swelling in the party might influence mother-offspring proximity, we coded the presence of females with a full sexual swelling in the party composition as "Yes" if there was at least one female with a full sexual swelling during the event, and "No" if there was not. We calculated the percentage of time spent with a female with a full sexual swelling per day of observation as the duration of observation when at least one female with a full sexual swelling was present in the party divided by the total duration of observation time per focal per day.

Detection of offspring not seeking comfort when showing vocal distress

In order to detect individual differences in comfort-seeking predicted by vocal distress, we examined the credible intervals for each individual (**Figure S3**). For each estimate per individual, we added the overall estimate of the effect (0.84) to the estimate of each individual for the random slope of the behavior of whimpering or screaming in **Model 1**. (**Table 1**). Point estimates of the effect were positive except for three individuals (SF27, EM9, and SF2) with a range extracted from random effect estimates of the random slopes varying between -0.21 and 2.44. We found that vocal distress did not predict an approach for three individuals. However, in events when there was no vocal distress, these offspring approached their mother in several cases.

Models for social and non-social exploration

A non-social context was defined by solitary play or exploration of the environment without interaction with another individual.

Model 4. For this analysis, we focused on a subset of 18 individuals aged 1.5 to 6 years, as attachment types were available exclusively for these individuals. To assess the effect of attachment type on the duration spent by the offspring exploring the environment in a non-social context per day, we calculated the percentage of time of non-social exploration (duration of non-social exploration per day/duration of total observation per day), we transformed the percentage into values between 0 and 1 excluding 0 to apply the Beta distribution ((percentage * (number of rows - 1) + 0.5) / number of rows). We used as the response variable this percentage corrected by the total duration of observation per day (using the weights function). Then we used as a test predictor the attachment type with an interaction with the age of the offspring and added the following control variables: (1) offspring's sex, (2) party size, (3) rank of the mother, (4) group, (5) percentage of time at least one female with full sexual swelling was present in the party. We did not control for the age of the mother since the VIFs value was above 5. The model explained 59% of the variance (R_2 = 0.593).

Model 5. Similar to **Model 4** but in a context of social exploration, during play bouts with other individuals than the mother. The model explained 65% of the variance (R_2 = 0.653).

We showed that there was a strong effect of the party size on the percentage of social and non-social exploration (**Tables S3**). The more individuals there were in the party, the more the individuals explored socially and not socially. There was also a strong effect of age on the percentage of non-social exploration. The percentage of non-social exploration decreased with age. There was no effect of the attachment types and the other variables on the percentage of social and non-social exploration.

Investigation of the latency time between a threat and exploration

Model 6. For this analysis, we focused on a subset of 18 individuals aged 1.5 to 6 years, as attachment types were available exclusively for these individuals. In order to assess the effect of the attachment type on the latency time between a threat and exploration, we used Bayesian estimation with a lognormal error distribution. We selected the threatening events as UTEs and threats during play bouts only when the mother approached her offspring to represent the offer

of comfort. Each threatening event constituted a data point and the response variable was the duration in seconds between a threatening event and a new explorative activity (social play, solitary play, exploration of the environment). Since the data collection is sometimes cut by bad observation of the individual's behavior, the duration was censored (using the function cens) by a variable name followed by exploration. If the behavior was constantly recorded without a cut (by bad observation or losing the individual), the value attributed was 0, if it was cut, we attributed the value 1. We used as a test predictor the attachment type with an interaction with the offspring's age. In addition, we included the following control variables since they might influence the offspring's behavior: (1) group, (2) offspring's sex, (3) party size, and (4) the offspring approaching the mother during the threat (1 if it occurred and 0 if not). The model explained 49% of the variance (R_2 = 0.487).

We showed that there was no difference in latency time between attachment types (**Table S4**). However, there was a strong effect of the party size, indeed, the more individuals there were in the party, the shorter time individuals took to explore after an UTE. The other variables did not have any effect on the latency time.

We ran all models in R 4.2.2[68] using the function 'brm' from the package 'brms'[70]. For all models, we included random intercepts for the identity of each immature offspring to account for repeated observations of the same individual while also pooling to account for average individual differences in the rates of the behavioral responses in each model[85]. Within these random intercepts, we also included random slopes for the age of the offspring and the party size for all models, the occurrence of whimpering or screaming by the offspring (**Model 1**), the percentage of time with a female with a full sexual swelling (**Models 3, 4 and 5**) to account for potential variations in the effects of age, party size, vocalizations, and sexual swelling on the response variables across different individuals. Random slopes allow for pooling to account for differences in the average responses of individuals to a given predictor variable[85]. The age of the offspring was calculated in months and the party size was calculated as the number of all individuals older than 10 years old in sight since the number of adult individuals might impact the offspring's behavior.

Additional information about the methods: UMAP, cluster analysis, and PCA

We performed the PCA using R Studio (R version 4.2.2)[68] and the UMAP and cluster analysis using Spyder Python (version 3.9)[69].

Behavioral patterns between individuals: Uniform Manifold Approximation and Projection

We used a recent technique for dimension reduction which allows data visualization similar to the dimension reduction technique 't-SNE' (t-distributed stochastic neighbor embedding): UMAP[86]. UMAP is a method for reducing the dimensionality of data while preserving its structure. It builds a graph connecting each data point to its nearest neighbors (the number of neighbors can be set up in the parameters) and assigns weights based on distances. By optimizing embedding to maintain local and global relationships, UMAP produces a lower-dimensional representation where similar points are close together and dissimilar ones are farther apart, making it useful for visualization and analysis. UMAP parameters were set such as number of components = 2 (indicating the number of dimensions represented), metric = Euclidean distance (calculating the straight-line distance between two points in Euclidean space), and learning rate = 1.0 (step size for the optimization process during embedding computation). We set up the minimum distance at 1 for better visual representation. We ensured reproducibility by fixing the initial random seed (random state) at 21 for UMAP. We found that using other values for the random state did not alter the outcome, reinforcing the reliability of our approach and giving the same result (respectively for random states equal 5 and 30 **Figure S4 (a) and (b)**). In order to find the best number of nearest neighbors, trials of different numbers from 2 to 5 were done with the expectation of showing the local structure of the data given the low number of individuals. Data structures and statistical computations were managed with 'Pandas'[87] and 'NumPy'[88]. All visualizations were generated using 'Matplotlib'[89].

Validity of the UMAP method: Cluster analysis

We performed a clustering analysis using 'Spectral Clustering' from 'scikit-learn'[90], to compare the results with the UMAP technique using a supervised method that requires specifying the number of clusters. Kernel spectral clustering with graph Laplacian regularization was the most suitable technique given the dataset[91,92]. Since we expected to have

three clusters corresponding to the three attachment types, the fact that spectral clustering does not suggest an optimal number of clusters was not an issue

Behavioral reactions to threat: Principal Component Analysis

To discern the driving behaviors behind each cluster, which are not given by the UMAP and the cluster analysis, we performed a PCA on the deviations of the behavioral reactions of the offspring (**Table 2 and Figure 5**). We conducted a parallel analysis using the function 'fa.parallel' from the 'psych' package[93] on the deviation estimates extracted from the Bayesian model explained above to determine the number of components needed for a PCA. Additionally, we performed a Kendall correlation test to check the collinearity between the behavioral variables using the function 'correlate' from the 'corr' package[94]. The highest collinearity coefficient was r = 0.778 between the variables "Approaching the mother" and "Looking towards the mother". Then, we performed a PCA on the deviation estimates using the 'prcomp' function from the 'R Base' package[95] on unscaled data in order to reduce the dimensionality while preserving important information. Visualizations were made using the 'ggplot2' package[96].

Figure S2: Individual differences in vocal distress predicting approaches: Credible intervals of Model 1

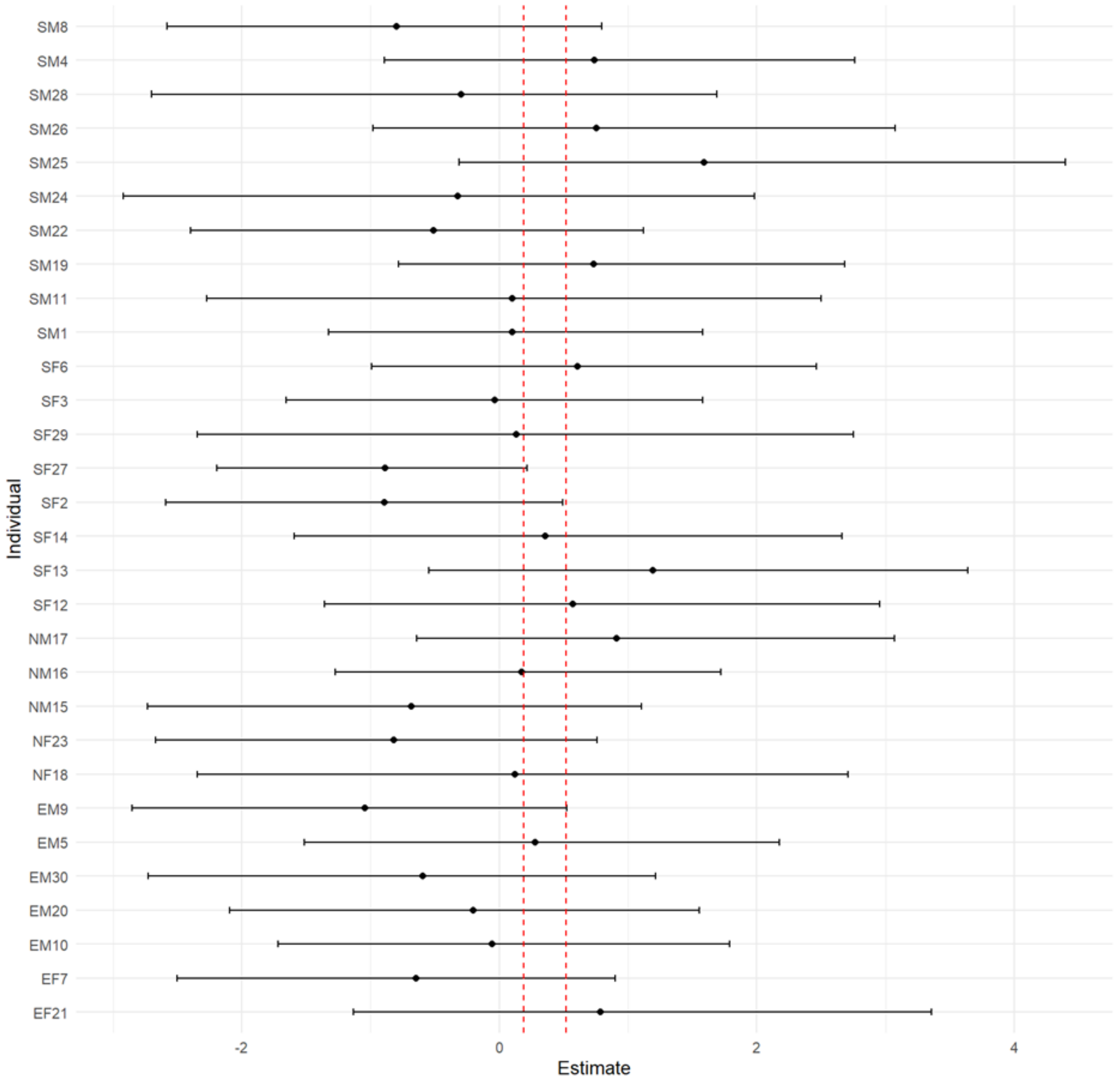

Figure S2. Representation of the credible interval for each offspring (in black) and for the overall model (in red) for the random slope of the behavior of whimpering or screaming in **Model 1**. Each dot represents an individual estimate for "Whimper or Scream" (random effect) for a specific individual. The horizontal lines around each dot represent the credible intervals (at 95%) for each individual's estimate. The effect of the model (vocal distress predicting approaches) does not apply to individuals whose credible intervals (in black) do not fully overlap with the credible interval of the overall model (in red). The sample size is N=30 individuals across 567 independent events.

Figure S3: Number of aggressions divided by total number of hours of observation per dyad mother-offspring

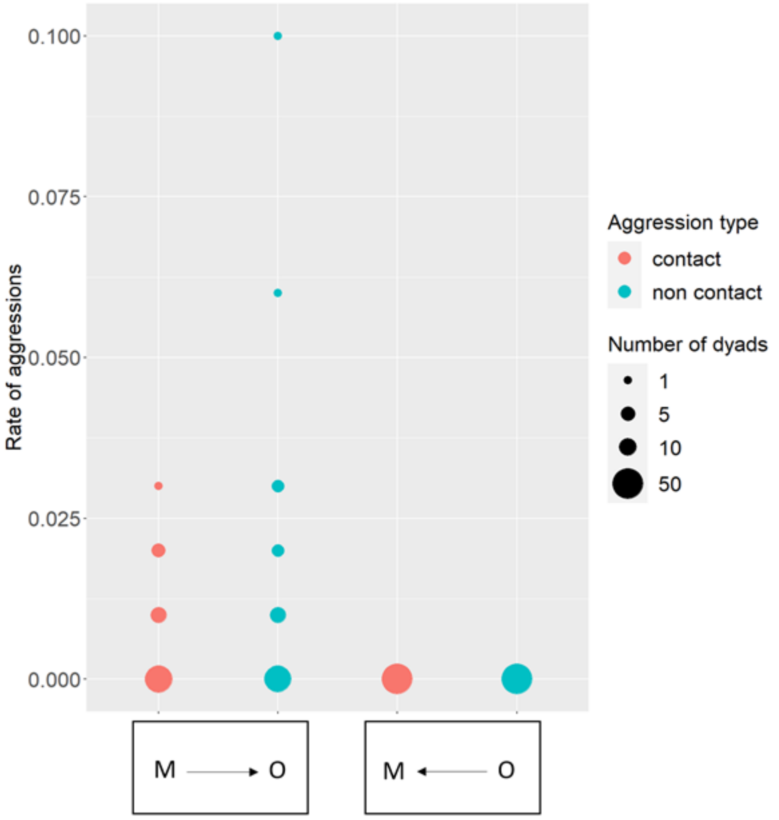

Figure S3. Contact and non-contact aggression rate per hour (indicated by the colors) given by the mother to the offspring or by the offspring to the mother (represented by the arrows, with M for mother and O for offspring). The sample size is N=50.

Figure S4: Dimension reduction (UMAP) and cluster analysis of the behavioral reactions of the offspring during undirected threatening events for different values of random state

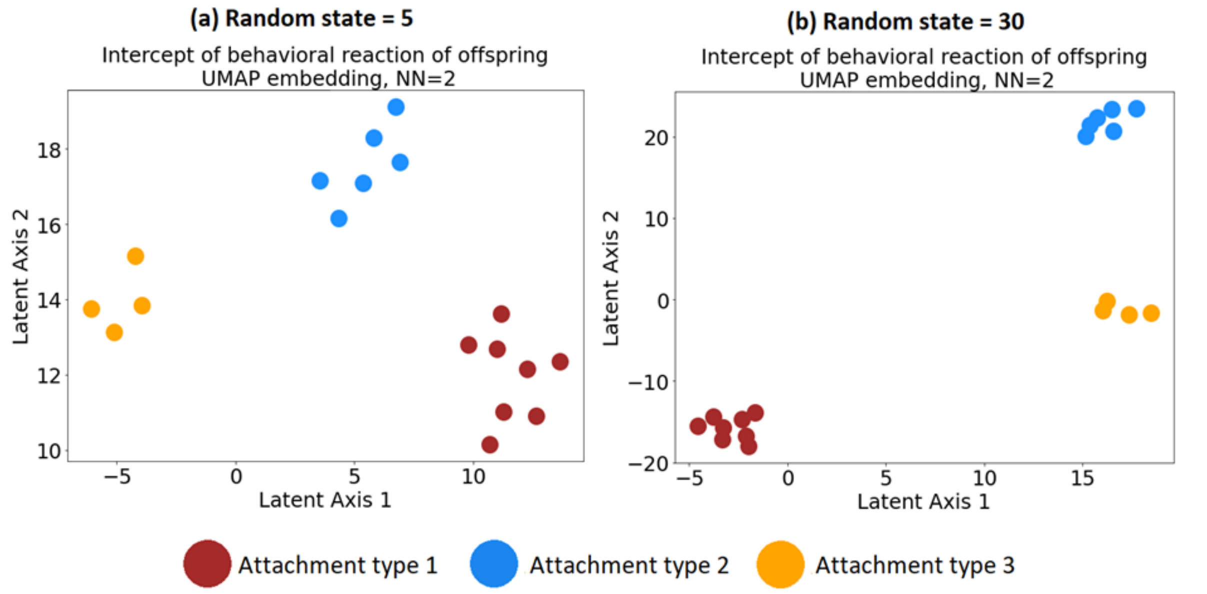

Figure S4. (a) Dimension reduction (UMAP) and cluster analysis random state = 5. (b) Dimension reduction (UMAP) and cluster analysis random state = 30. Each data point corresponds to an individual offspring. The position of the points corresponds to the UMAP representation, while the colors attributed correspond to the spectral cluster analysis. The axis of both plots represents a dimension reduction of the original data to two dimensions. The sample size is N=18.

<u>Table S3: Attachment type not predicting the percentage of non-social and social exploration: the result of Models 4 and 5</u>

	Estimate	Estimate error	95% CI	89% CI
**Non-social exploration**				
**Intercept**	9.07	1.69	[5.69 - 12.31]	[6.31 - 11.71]
**AT1 - AT3**	0.08	0.90	[-1.65 - 1.82]	[-1.35 - 1.52]
**AT2 - AT3**	-0.65	0.92	[-2.40 - 1.17]	[-2.09 - 0.82]
**AT2 - AT1**	0.73	1.21	[-1.71 - 3.03]	[-1.22 - 2.65]
**Offspring age**	-1.53	0.90	[-3.22 - 0.23]	**[-2.94 - -0.09]**
**Percentage swelling**	-0.06	0.49	[-1.01 - 0.89]	-0.84; 0.73]
**Sex**	-0.32	0.91	[-2.13; 1.51]	[-1.77; 1.15]
**Group South**	-0.10	0.91	[-1.91; 1.73]	[-1.53; 1.41]
**Group East**	0.07	0.90	[-1.79; 1.67]	[-1.50; 1.36]
**Mother rank**	-0.26	0.70	[-1.60; 1.12]	[-1.36; 0.87]
**Party size**	1.29	0.57	**[0.13; 2.36]**	**[0.35; 2.16]**
**AT1 : Offspring age - AT3 : Offspring age**	-1.00	0.88	[-2.69; 0.75]	[-2.39; 0.42]
**AT2 : Offspring age AT3 : Offspring age**	0.11	0.93	[-1.70; 1.92]	[-1.39; 1.61]
**AT2 : Offspring age – AT1 : Offspring age**	-0.35	1.25	[-2.81; 2.08]	[-2.37; 1.62]
**Social exploration**				
**Intercept**	9.75	1.38	[7.02; 12.54]	[7.53; 11.99]
**AT1 1 - AT3**	-0.71	0.87	[-2.41; 1.02]	[-2.09; 0.68]
**AT2 - AT3**	0.51	0.89	[-1.22; 2.22]	[-0.94; 1.93]
**AT2 - AT1**	-1.22	1.16	[-3.48; 1.05]	[-3.08; 0.64]
**Offspring age**	-0.70	0.73	[-2.15; 0.72]	[-1.85; 0.45]
**Percentage swelling**	0.18	0.60	[-0.99; 1.32]	[-0.77; 1.12]
**Sex**	0.34	0.89	[-1.40; 2.07]	[-1.07; 1.76]
**Group South**	0.24	0.88	[-0.49; 0.93]	[-1.21; 1.61]
**Group East**	0.19	0.89	[-1.59; 1.88]	[-1.28; 1.58]
**Mother rank**	-0.55	0.66	[-1.83; 0.75]	[-1.60; 0.51]
**Party size**	2.35	0.68	**[0.87; 3.59]**	**[1.19; 3.39]**
**AT1 : Offspring age - AT3 : Offspring age**	-0.08	0.82	[-1.70; 1.56]	[-1.36; 1.23]
**AT2 : Offspring age AT3 : Offspring age**	-0.06	0.89	[-1.82; 1.69]	[-1.50; 1.35]
**AT2 : Offspring age – AT1 : Offspring age**	-0.59	1.18	[-2.90; 1.74]	[-2.46; 1.32]

AT1, AT2, and AT3 correspond to attachment type 1, attachment type 2, and attachment type 3. 95% CI and 89% CI are the credible intervals at their confidence levels, respectively. The reference levels for each predictor are

female for “Sex”, and North for “Group”. Numbers in bold represent credible intervals excluding 0. The sample size is N=18.

Table S4: Attachment type not predicting latency time between a threat and exploration: the results of Model 6

	Estimate	Estimate error	95% CI	89% CI
**Intercept**	5.72	1.06	[3.54; 7.78]	[4.00; 7.35]
**AT1 - AT3**	0.68	0.98	[-1.27; 2.58]	[-0.88; 2.21]
**AT2 - AT3**	0.54	1.07	[-1.48; 2.73]	[-1.09; 2.27]
**AT2 - AT1**	0.14	1.17	[-2.25; 2.39]	[-1.73; 1.96]
**Offspring age**	-0.13	0.90	[-1.99; 1.61]	[-1.58; 1.25]
**Sex**	-0.15	0.93	[-2.07; 1.68]	[-1.64; 1.29]
**Approach by offspring**	-0.13	0.41	[-0.92; 0.69]	[-0.79; 0.53]
**Group South**	-0.55	0.94	[-2.41; 1.42]	[-1.98; 1.00]
**Group East**	-1.01	1.14	[-3.17; 1.46]	[-2.73; 0.87]
**Party size**	-0.38	0.23	[-0.83; 0.07]	**[-0.74; -0.02]**
**AT1 : Offspring age - AT3 : Offspring age**	1.08	1.51	[-2.03; 4.05]	[-1.33; 3.44]
**AT2 : Offspring age AT3 : Offspring age**	-1.85	2.83	[-7.47; 3.78]	[-6.33; 2.59]
**AT2 : Offspring's age – AT1 : Offspring age**	2.94	3.15	[-3.27; 9.21]	[-2.07; 7.96]

AT1, AT2, and AT3 correspond to attachment type 1, attachment type 2, and attachment type 3. 95% CI and 89% CI are the credible intervals at their confidence levels, respectively. The reference levels for each predictor are female for “Sex”, and North for “Group”. Numbers in bold represent credible intervals excluding 0. The sample size is N=18.

Figure S5: Posterior predictive checks for Model 2 (behavioral reactions of offspring during threatening events)

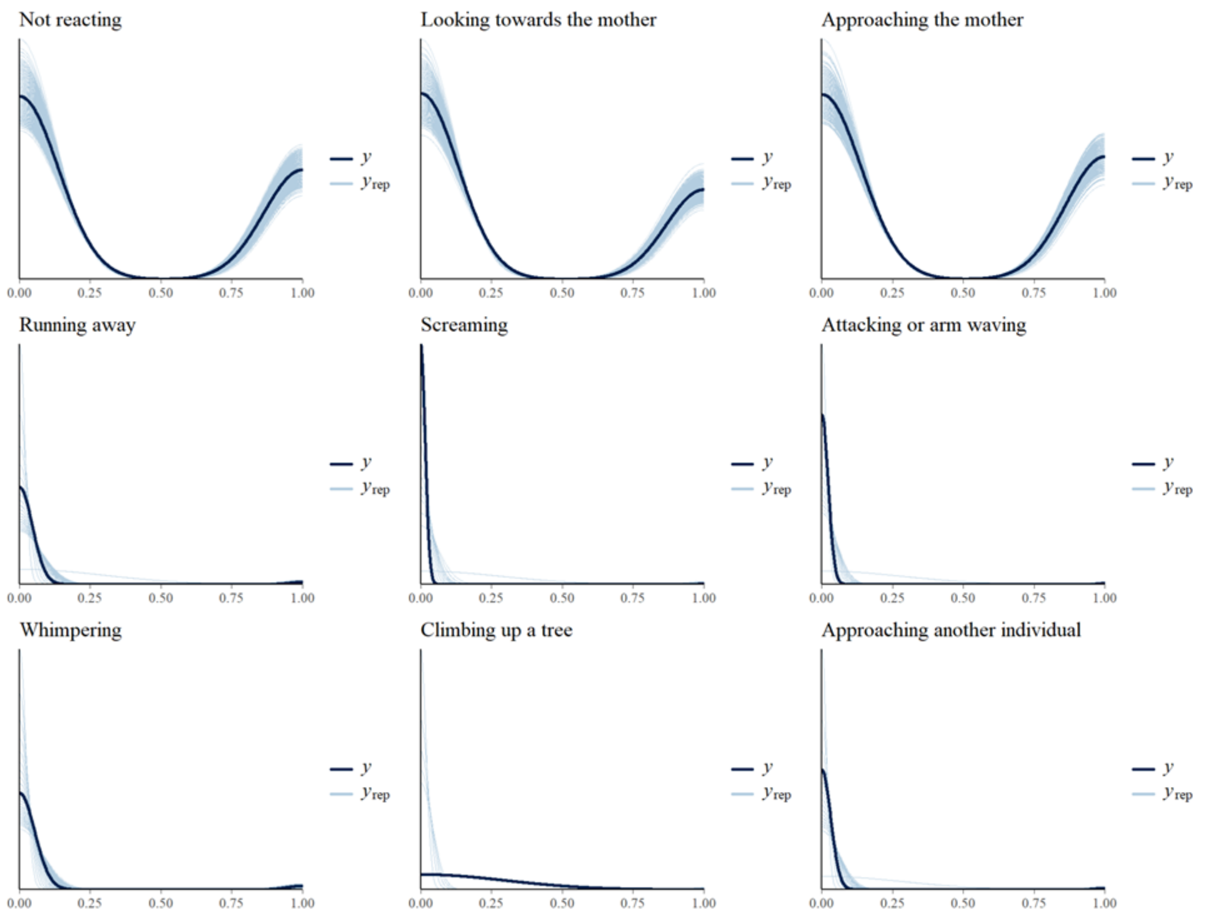

The black line (y) represents the observed data distribution and the blue lines (y rep) represent the multiple simulated posterior predictive distributions.

Figure S6: Posterior predictive checks for Models 1, 3, 4, 5, and 6

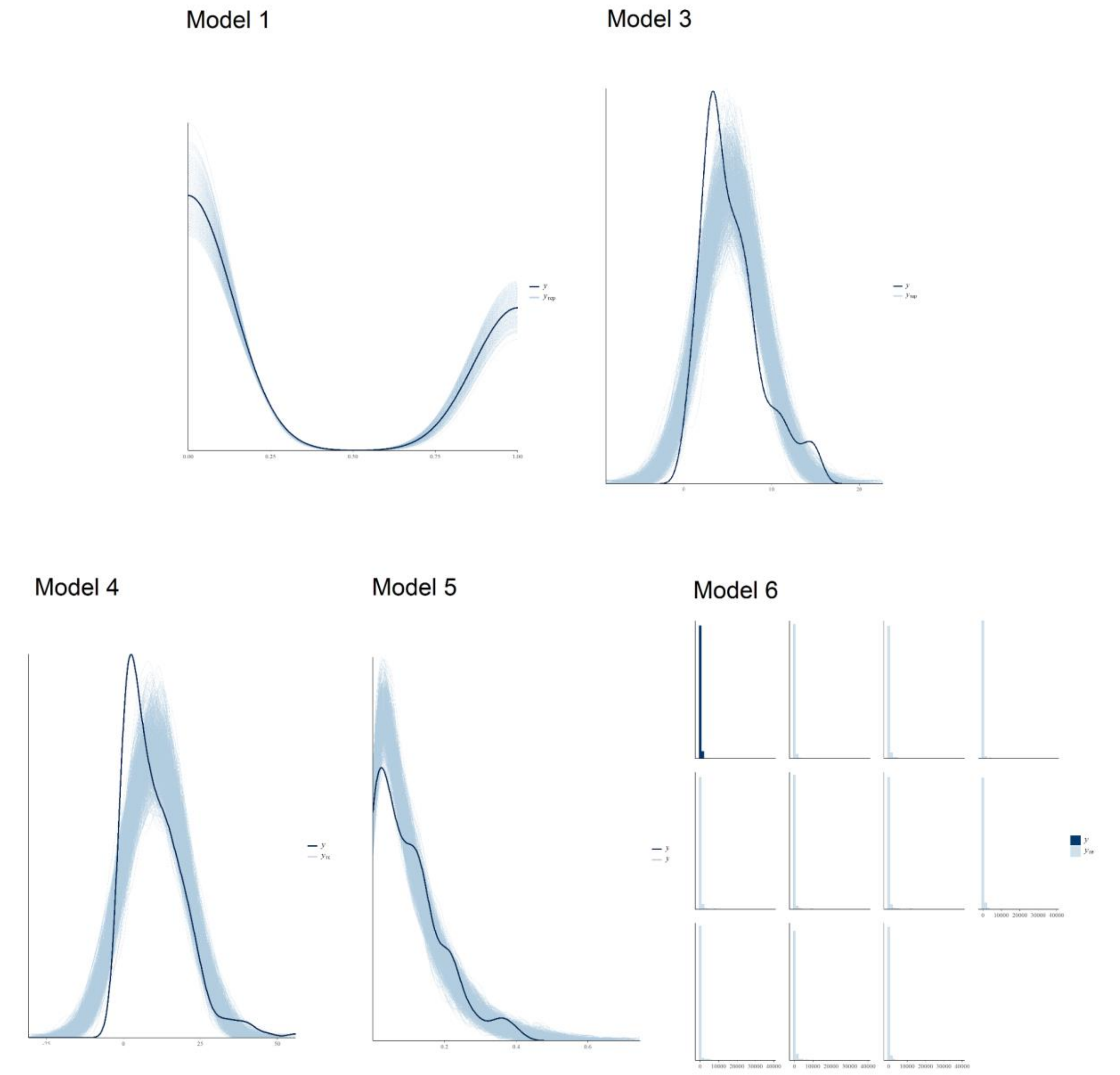

For models 1, 3, 4, and 5, the black line (y) represents the observed data distribution and the blue lines (y rep) represent the multiple simulated posterior predictive distributions. For Model 6, the black bar (y) represents the observed data distribution and the blue bars (y rep) represent the multiple simulated posterior predictive distributions.

74. Pusey, A. E. (1990). Behavioural changes at adolescence in chimpanzees. Behaviour, 115(3-4), 203-246.

75. Lonsdorf, E. V., Markham, A. C., Heintz, M. R., Anderson, K. E., Ciuk, D. J., Goodall, J., & Murray, C. M. (2014). Sex differences in wild chimpanzee behavior emerge during infancy. PLoS One, 9(6), e99099.

76. White, F. J. (1992). Pygmy chimpanzee social organization: variation with party size and between study sites. American Journal of Primatology, 26(3), 203-214.

77. Stewart, R. B. (1983). Sibling attachment relationships: Child–infant interaction in the strange situation. Developmental psychology, 19(2), 192.

78. Herrera, A., & Zuberbühler, P. K. (2017). Mother-Offspring Spatial Relationships in Wild Eastern Chimpanzees (Pan troglodytes schweinfurthii).

79. Muller, M. N., & Wrangham, R. W. (2004). Dominance, aggression, and testosterone in wild chimpanzees: a test of the ‘challenge hypothesis’. Animal behavior, 67(1), 113-123.

80. Preis, A. N. N. A., Samuni, L., Deschner, T., Crockford, C., & Wittig, R. O. M. A. N. (2019). Group-specific social dynamics affect urinary oxytocin levels in Taï male chimpanzees. The chimpanzees of the Taï forest, 40.

81. Wittig, R. M., & Boesch, C. (2003) (b). Food competition and linear dominance hierarchy among female chimpanzees of the Tai National Park. International Journal of Primatology, 24, 847-867.

82. Neumann, C., Duboscq, J., Dubuc, C., Ginting, A., Irwan, A. M., Agil, M., ... & Engelhardt, A. (2011). Assessing dominance hierarchies: validation and advantages of progressive evaluation with Elo-rating. Animal Behaviour, 82(4), 911-921.

83. Foerster, J., Assael, I. A., De Freitas, N., & Whiteson, S. (2016). Learning to communicate with deep multi-agent reinforcement learning. In Advances in Neural Information Processing Systems, 29.

84. Mielke, A., Samuni, L., Preis, A., Gogarten, J. F., Crockford, C., & Wittig, R. M. (2017). Bystanders intervene to impede grooming in Western chimpanzees and sooty mangabeys. Royal Society open science, 4(11), 171296.

85. Barr, D. J., Levy, R., Scheepers, C., & Tily, H. J. (2013). Random effects structure for confirmatory hypothesis testing: Keep it maximal. Journal of memory and language, 68(3), 255-278.

86. McInnes, L., Healy, J., & Melville, J. (2018). Umap: Uniform manifold approximation and projection for dimension reduction. arXiv preprint arXiv:1802.03426.

87. McKinney, W. (2010). Data structures for statistical computing in Python. Proceedings of the 9th Python in Science Conference, 56, 51-56.

88. Harris, C. R., Millman, K. J., van der Walt, S. J., Gommers, R., Virtanen, P., Cournapeau, D., Heckel, A., Gohlke, C., & Oliphant, T. E. (2020). Array programming with NumPy. Nature, 585(7825), 357–362.

89. Hunter, J. D. (2007). Matplotlib: A 2D graphics environment. Computing in Science & Engineering, 9(3), 90-95.

90. Pedregosa, F., Varoquaux, G., Gramfort, A., Michel, V., Thirion, B., Grisel, O., ... & Duchesnay, E. (2011). Scikit-learn: Machine learning in Python. Journal of Machine Learning Research, 12, 2825-2830.

91. Rodriguez, M. Z., Comin, C. H., Casanova, D., Bruno, O. M., Amancio, D. R., Costa, L. D. F., & Rodrigues, F. A. (2019). Clustering algorithms: A comparative approach. PloS one, 14(1), e0210236.

92. Murugesan, N., Cho, I., & Tortora, C. (2021). Benchmarking in cluster analysis: a study on spectral clustering, DBSCAN, and K-Means. In Data Analysis and Rationality in a Complex World 16 (pp. 175-185). Springer International Publishing.

93. Horn, J. L. (1965). A rationale and test for the number of factors in factor analysis. Psychometrika, 30, 179-185.

94. Chang, W., & Xie, Y. (2020). corrr: Exploratory Correlation Analysis in R. R package version 0.4.6.

95. Holland, S. M. (2008). Principal components analysis (PCA). Department of Geology, University of Georgia, Athens, GA, 30602, 2501.

96. Wickham, H. (2016). ggplot2: Elegant Graphics for Data Analysis. Springer-Verlag New York.
