# Supplementary material for: Evidence of organized but not disorganized attachment in wild Western chimpanzee offspring (Pan troglodytes verus)
Source: Nat Hum Behav. 2025 May 12;9(8):1571–82. doi: 10.1038/s41562-025-02176-8 (PMC12367522; doi:10.1038/s41562-025-02176-8)
Supplement: Supplementary file 2 — Reporting Summary [file 41562_2025_2176_MOESM2_ESM.pdf]

nature portfolio

DAPR
NATHUMBEHAV-24062231
Corresponding author(s): Eléonore Rolland and Roman Wittig

Last updated by author(s): Feb 7, 2025

Reporting Summary

Nature Portfolio wishes to improve the reproducibility of the work that we publish. This form provides structure for consistency and transparency in reporting. For further information on Nature Portfolio policies, see our Editorial Policies and the Editorial Policy Checklist.

Statistics

For all statistical analyses, confirm that the following items are present in the figure legend, table legend, main text, or Methods section.

n/a	Confirmed	
☐	☒	The exact sample size (n) for each experimental group/condition, given as a discrete number and unit of measurement
☐	☒	A statement on whether measurements were taken from distinct samples or whether the same sample was measured repeatedly
☐	☒	The statistical test(s) used AND whether they are one- or two-sided<br>*Only common tests should be described solely by name; describe more complex techniques in the Methods section.*
☐	☒	A description of all covariates tested
☐	☒	A description of any assumptions or corrections, such as tests of normality and adjustment for multiple comparisons
☐	☒	A full description of the statistical parameters including central tendency (e.g. means) or other basic estimates (e.g. regression coefficient) AND variation (e.g. standard deviation) or associated estimates of uncertainty (e.g. confidence intervals)
☒	☐	For null hypothesis testing, the test statistic (e.g. F, t, r) with confidence intervals, effect sizes, degrees of freedom and P value noted<br>*Give P values as exact values whenever suitable.*
☐	☒	For Bayesian analysis, information on the choice of priors and Markov chain Monte Carlo settings
☒	☐	For hierarchical and complex designs, identification of the appropriate level for tests and full reporting of outcomes
☒	☐	Estimates of effect sizes (e.g. Cohen's d, Pearson's r), indicating how they were calculated

Our web collection on statistics for biologists contains articles on many of the points above.

Software and code

Policy information about availability of computer code

Data collection	Cybertracker (version 3.517)
Data analysis	R Studio (version 4.2.2), Spyder Python (version 3.9)

For manuscripts utilizing custom algorithms or software that are central to the research but not yet described in published literature, software must be made available to editors and reviewers. We strongly encourage code deposition in a community repository (e.g. GitHub). See the Nature Portfolio guidelines for submitting code & software for further information.

Data

Policy information about availability of data

All manuscripts must include a data availability statement. This statement should provide the following information, where applicable:

- Accession codes, unique identifiers, or web links for publicly available datasets
- A description of any restrictions on data availability
- For clinical datasets or third party data, please ensure that the statement adheres to our policy

All data for the analyses supporting the findings of this study are available within the paper and its Supplementary Information.

Research involving human participants, their data, or biological material

Policy information about studies with human participants or human data. See also policy information about sex, gender (identity/presentation), and sexual orientation and race, ethnicity and racism.

Reporting on sex and gender	N/A
Reporting on race, ethnicity, or other socially relevant groupings	N/A
Population characteristics	N/A
Recruitment	N/A
Ethics oversight	N/A

Note that full information on the approval of the study protocol must also be provided in the manuscript.

Field-specific reporting

Please select the one below that is the best fit for your research. If you are not sure, read the appropriate sections before making your selection.

☐ Life sciences ☒ Behavioural & social sciences ☐ Ecological, evolutionary & environmental sciences

For a reference copy of the document with all sections, see nature.com/documents/nr-reporting-summary-flat.pdf

Behavioural & social sciences study design

All studies must disclose on these points even when the disclosure is negative.

Study description	We collected behavioural data on wild chimpanzees. The data were a mix between qualitative and quantitative.
Research sample	Data was collected on three wild Western chimpanzee (P. t. verus) communities (North, South, and East Group), habituated to human observers and located in the Taï National Park, Côte d'Ivoire. Individual's age varied between 0 and 10 years old gathering 20 females and 30 males. This sample was selected to investigate mother-offspring attachment, as attachment develops in the early years of life, and in chimpanzees, maternal dependence extends throughout the first decade. While every effort was made to achieve a balanced sex ratio, field conditions and the limited number of habituated individuals constrained equal representation of males and females. Nonetheless, the sample is considered representative for examining attachment patterns in wild chimpanzees.
Sampling strategy	The sampling procedure was "convenience". We focused on young chimpanzees because attachment type is the best shown in young individuals due to high frequency of mother-offspring interactions. Given that we worked in a wild environment, we were limited in the number of individuals and worked on 3 chimpanzee communities gathering a total of 50 individuals. We believe 50 individuals is enough to investigate the existence of disorganized attachment. We used a subset of the data (18 individuals ages 1.5-6 years old) to investigate the existence of distinct attachment types. This age range was chosen because it allows the observation of potential differences in attachment security between mother and offspring, which is not possible or very difficult outside of this age range. With 18 individuals, we observed individual difference in attachment types. Addiotnally, we observed multiple threatening situations per individuals, contrary to the studies on attachment types conducted in humans (Strange Situation Procedure) which conducted the experiment only once per individual. Data saturation was considered. Indeed, we conducted of pilot study of 8 months before the data collection to ensure all possible behaviours were encoded and we collected a minimum of 50 hours of observation per individual.
Data collection	The data were collected using the sofware Cybertracker installed on a phone device. Multiple observers could be present observing the group (4 maximum) and each one was collecting data on one respective individual.
Timing	Data were collected from December 2016 to June 2018 on 27 immature chimpanzees, and from December 2021 to April 2023 on 30 immature chimpanzees. Seven individuals were present in both datasets totalling 50 individuals over the two time periods of ata collection.
Data exclusions	No data were excluded from the analysis.
Non-participation	No participant declined participation.
Randomization	There was no experimental group.

Reporting for specific materials, systems and methods

We require information from authors about some types of materials, experimental systems and methods used in many studies. Here, indicate whether each material, system or method listed is relevant to your study. If you are not sure if a list item applies to your research, read the appropriate section before selecting a response.

Materials & experimental systems

n/a	Involved in the study
☒	☐ Antibodies
☒	☐ Eukaryotic cell lines
☒	☐ Palaeontology and archaeology
☐	☒ Animals and other organisms
☒	☐ Clinical data
☒	☐ Dual use research of concern
☒	☐ Plants

Methods

n/a	Involved in the study
☒	☐ ChIP-seq
☒	☐ Flow cytometry
☒	☐ MRI-based neuroimaging

Animals and other research organisms

Policy information about studies involving animals; ARRIVE guidelines recommended for reporting animal research, and Sex and Gender in Research

Laboratory animals	This study did not involve laboratory animals.
Wild animals	We collected behavioural data on three communities of wild western chimpanzees (Pan troglodytes verus) in Taï National Park (Côte d'Ivoire) habituated to human observers. We followed the strict hygiene protocol of the Taï Chimpanzee Project, adopted by IUCN as the best practice guideline for wild ape studies. No animal was captured and the study was purely observational and non-invasive. The animals were between 0 and 10 years old.
Reporting on sex	Sex was assigned using visual information of genital organs. We included the sex of the individuals as a control variables in our models. There was no effect of sex in our analyses.
Field-collected samples	This study did not involve samples collected from the field.
Ethics oversight	The research protocol used was approved by the 'Ethikrat' of the Max Planck Society on 04.08.2014 and by the Directeur de Recherche en Cote d'Ivoire under Permit TCP 008-/MESRS/DGRI from 5.5.2021.

Note that full information on the approval of the study protocol must also be provided in the manuscript.

Plants

Seed stocks	N/A
Novel plant genotypes	N/A
Authentication	N/A
